# Sex specific regulation of TSPY-Like 2 in the DNA damage response of cancer cells

**DOI:** 10.1038/s41419-023-05722-2

**Published:** 2023-03-15

**Authors:** Miriana Cardano, Martina Magni, Roberta Alfieri, Siu Yuen Chan, Simone Sabbioneda, Giacomo Buscemi, Laura Zannini

**Affiliations:** 1 Istituto di Genetica Molecolare, Consiglio Nazionale delle Ricerche (IGM-CNR), 27100 Pavia, Italy; 2 Department of Medical Oncology and Hematology, Fondazione IRCCS Istituto Nazionale dei Tumori, Via Venezian 1, 20133 Milan, Italy; 3 Department of Paediatrics and Adolescent Medicine, The University of Hong-Kong, Hong-Kong SAR, China

**Keywords:** DNA damage response, Stress signalling

## Abstract

Females have a lower probability to develop somatic cancers and a better response to chemotherapy than males. However, the reasons for these differences are still not well understood. The X-linked gene TSPY-Like 2 (TSPYL2) encodes for a putative tumor suppressor protein involved in cell cycle regulation and DNA damage response (DDR) pathways. Here, we demonstrate that in unstressed conditions TSPYL2 is maintained at low levels by MDM2-dependent ubiquitination and proteasome degradation. Upon genotoxic stress, E2F1 promotes *TSPYL2* expression and protein accumulation in non-transformed cell lines. Conversely, in cancer cells, TSPYL2 accumulates only in females or in those male cancer cells that lost the Y-chromosome during the oncogenic process. Hence, we demonstrate that while TSPYL2 mRNA is induced in all the tested tumor cell lines after DNA damage, TSPYL2 protein stability is increased only in female cancer cells. Indeed, we found that TSPYL2 accumulation, in male cancer cells, is prevented by the Y-encoded protein SRY, which modulates MDM2 protein levels. In addition, we demonstrated that TSPYL2 accumulation is required to sustain cell growth arrest after DNA damage, possibly contributing to protect normal and female cancer cells from tumor progression. Accordingly, TSPYL2 has been found more frequently mutated in female-specific cancers. These findings demonstrate for the first time a sex-specific regulation of TSPYL2 in the DDR of cancer cells and confirm the existence of sexual dimorphism in DNA surveillance pathways.

## Introduction

Sexual dimorphism has been demonstrated to play a critical role in the incidence and survival of cancer types unrelated to reproductive functions. Indeed, females show a better prognosis and a lower risk to develop cancers unrelated to sex than males [1, 2], but reasons for these differences are still unclear.

The DNA damage response (DDR) is a network that efficiently coordinates mechanisms such as DNA repair, apoptosis, and cell cycle regulation aimed at maintaining genome stability [3]. DDR defects have been associated with cancer development, but, at the same time, they represent a weakness for cancer cells, which can be exploited by anticancer therapies, since these cells are more sensitive to DNA-damaging drugs than normal cells [4]. At present, the role of sexual dimorphism in the DDR is an emerging field of study and many aspects remain unknown [5].

Testis-specific protein Y-encoded-like 2 (TSPYL2, also named DENTT, CDA1 and TSPX) is a nuclear protein of the TSPY-L nucleosome assembly protein 1 super-family characterized by the presence of a predicted nucleosome assembly protein (NAP) domain necessary for chromatin remodeling and gene expression regulation [6]. It is encoded by an X-linked gene, and has a homologue on the Y chromosome, TSPY, which has been found overexpressed in different cancer types and described as a proto-oncogene [7, 8]. TSPYL2 is known to be involved in the regulation of cell cycle progression [9] and DDR pathways [10, 11]. Moreover, we recently demonstrated that TSPYL2 regulates p53-dependent apoptosis after DNA damage by repressing and promoting respectively the activity of SIRT1 deacetylase and p300 acetyltransferase [12]. TSPYL2 has been found to be mutated in endometrial carcinoma [13] and downregulated in glioma [14, 15], human and mouse lung primary tumors [16] and hepatocellular carcinoma [17]. These data together with the finding that TSPYL2 overexpression in lung and breast cancer reduces cell growth and the migratory potential [16], suggest for this protein a tumor suppressor role.

The transcription factor E2F1 is a crucial player in several cellular pathways, including DNA replication, cell cycle regulation and apoptosis induction [18]. The major regulator of E2F1 is the tumor suppressor protein retinoblastoma (pRB). When pRB is hypophosphorylated, it binds to E2F1 repressing its transcriptional activity finally suppressing G1/S transition. When hyperphosphorylated, pRB is incapable of binding E2F1, which is free to promote the transcription of target genes and cell cycle progression [19]. Importantly, the pRB-E2F1 pathway has been recently associated to sex disparities found in glioma, which affects more males than females [20, 21].

Sex-determining region Y (SRY) is a transcription factor encoded by a gene located in the male-specific Y chromosome and belonging to the family of Sox proteins [22]. SRY is normally expressed during embryogenesis to promote male sex determination, while in adult tissues it is completely silenced by DNA hypermethylation, except for the brain, thymus and testis. Deregulated expression of SRY has been found in male tumors where it demonstrated oncogenic properties, possibly contributing to the sexual dimorphism present in certain types of cancer [23, 24].

Here, we demonstrate, for the first time, that, after DNA damage, TSPYL2 protein is regulated in a sex-specific manner in cancer cells. Indeed, in unstressed conditions, TSPYL2 is maintained at low levels by ubiquitin ligase murine double minute 2 (MDM2)-dependent degradation. After DNA damage, E2F1 promotes *TSPYL2* gene expression in cells of both sexes, but the protein accumulates only in female cancer cells, where it is no longer ubiquitinated. Conversely, in male cancer cells, TSPYL2 is still ubiquitinated by MDM2 and degraded. These disparities are caused by the expression of SRY that modulates MDM2 protein levels. Finally, we found that TSPYL2 accumulation is required to prevent the proliferation of female cancer cells exposed to DNA damage and that, accordingly, *TSPYL2* gene is more frequently mutated in female-specific tumors.

## Results

### TSPYL2 accumulates in normal and in female cancer cells after DNA damage

**Fig. 1:**
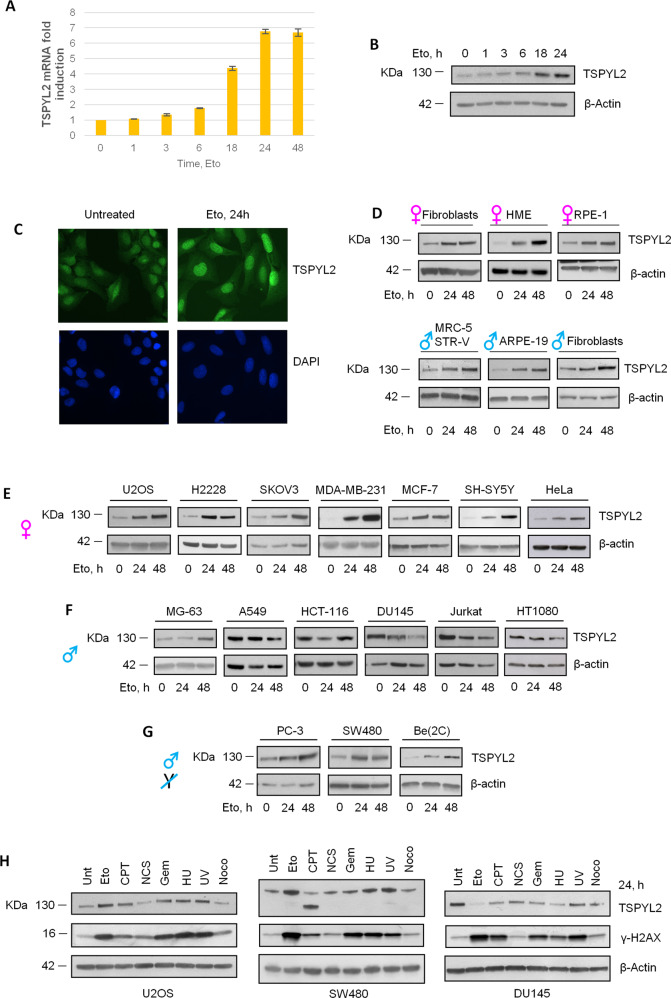
TSPYL2 mRNA and protein are induced in response to DNA damage in normal and female cancer cell lines. **A** RT-qPCR analysis of TSPYL2 accumulation in U2OS cells upon 1, 3, 6, 18, 24 and 48 h of etoposide treatment. **B** Western blot analysis of TSPYL2 levels in U2OS cells in untreated condition and after 1, 3, 6, 18 and 24 h of etoposide. **C** Immunofluorescence analysis of TSPYL2 levels after etoposide treatment in U2OS cells. **D** Western blot analysis of TSPYL2 levels upon 24 h and 48 h of etoposide treatment in untransformed cell lines, **E** female cancer cell lines, **F** male cancer cell lines and **G** male cancer cell lines that lost the Y chromosome. **H** Western blot analysis of TSPYL2 and γH2AX levels in U2OS, SW480 and DU145 cells treated with different types of genotoxic agents for 24 h. Eto etoposide, CPT camptothecin, NCS neocarzinostatin, Gem gemcitabine, HU hydroxyurea, UV ultraviolet radiation, NOCO nocodazole.

To deeply investigate TSPYL2 role in the DDR, we performed a time course analysis in the osteosarcoma cell line U2OS in response to 20 μM treatment with etoposide, a topoisomerase II inhibitor [25]. Induction of DNA damage was verified evaluating γ-H2AX and p53 accumulation [25], while apoptosis was tested by investigating PARP-1 cleavage (Supplementary Fig. 1A). Analyses of TSPYL2 revealed an induction at both mRNA and protein levels starting from 3–6 h to, at least, 24 h of etoposide treatment (Fig. 1A and B) and protein accumulation in the nucleus of the treated cells (Fig. 1C and Supplementary Fig. 1B).

To extend our findings, we investigated TSPYL2 protein levels 24 and 48 h after etoposide treatment in different human non-transformed and cancer cell lines (Table 1). We found that 24 h after drug administration TSPYL2 accumulates in all the tested non-transformed cells and its levels further increased at 48 h (Fig. 1D). These results clearly indicate that at late time points after DNA damage TSPYL2 protein is commonly gradually induced in nontransformed cells.

**Table 1:** Non-transformed and cancer cell line.

NON-TRANSFORMED CELL LINES	MALE	FEMALE
Skin fibroblasts	Primary skin fibroblasts	Primary skin fibroblasts
Normal retina epithelium	ARPE-19	RPE-1
Normal lung fibroblasts	MRC-5 STRV	
Normal mammary epithelium		HME-1
**CANCER CELL LINES**	**MALE**	**FEMALE**
Osteosarcoma	MG-63	U2OS
Lung cancer	A549	H2228
Neuroblastoma	Be(2)-C (NO Y)	SH-SY5Y
Colon cancer	HCT-116	
Colon cancer	SW480 (NO Y)	
T-cell Leukemia	Jurkat	
Fibrosarcoma	HT1080	
Prostate cancer	DU145	
Prostate cancer	PC-3 (NO Y)	
Ovarian cancer		SKOV3
Breast cancer		MDA-MB-231
Breast cancer		MCF-7
Cervical carcinoma		HeLa

The nontransformed and cancer-human cell lines used in these studies are listed in the table. The tissue of origin and sex are indicated.

A different behavior was instead interestingly observed in cancer cells. Like non-transformed cell lines, TSPYL2 levels raised at 24 and 48 h after etoposide treatment in female cancer cells (Fig. 1E), while on the contrary, a more variable regulation, never leading to the protein accumulation found in normal cells, was detected in male cells. Indeed, some male cancer cell lines showed a reduction of TSPYL2 after etoposide (e.g. A549, DU145, Jurkat and HT1080), while in others (e.g. MG-63 and HCT116) TSPYL2 decreased at 24 h after damage and then accumulated at 48 h (Fig. 1F). Strikingly, we also found that upon etoposide, TSPYL2 protein is induced in those male cancer cells (e.g. PC3, SW480 and Be2C) that, during the oncogenic process, lost the Y chromosome [26] (Fig. 1G).

Importantly, as demonstrated by proliferation and cell cycle profile analyses (Supplementary Fig. 1C-D), the variable TSPYL2 regulations are not due to significant differences in cell growth among the cell lines, nor to disparities in the physiological levels of this protein since, in untreated cancer cells, TSPYL2 exhibits sex-independent variable levels (Supplementary Fig. 1E).

Altogether these data indicate that, after etoposide treatment, TSPYL2 protein accumulates in normal and in female cancer cell lines, but not in male cancer cells, unless they lost the Y chromosome.

To investigate the DNA damage specificity of TSPYL2 accumulation, we treated U2OS (female), DU145 (male) and SW480 (Y negative male) cells with different genotoxic agents (Table 2) and the presence of DNA lesions was determined by γ-H2AX levels evaluation (Fig. 1H). We found that, while no treatment promoted TSPYL2 accumulation in the male DU145 cell line, all the tested genotoxic agents, except for neocarzinostatin and nocodazole, and gemcitabine for SW480, caused TSPYL2 induction in U2OS and SW480 (Fig. 1H). Of note, camptothecin treatment accumulated a shorter form of TSPYL2 in SW480 cells, possibly the result of a splicing event.

**Table 2:** Genotoxic agents and their action.

DRUG	ACTIVITY
Etoposide (Eto)	Topoisomerase II inhibitor
Neocarzinostatin (NCS)	Radiomimetic drug
UV	Pyrimidine dimers formation
Gemcitabine (Gem)	DNA synthesis inhibitor
Hydroxyurea (HU)	Ribonucleotide reductase inhibitor
Camptothecin (CPT)	Topoisomerase I inhibitor
Nocodazole (Noco)	Microtubules depolarization

Genotoxic agents tested in these experiments and their action are reported in the table.

These data may suggest that TSPYL2 induction is fostered by genotoxic agents that cause double-strand breaks (DSBs) as secondary events, mostly during S-phase progression.

To verify this, we simultaneously treated U2OS cells with etoposide and EdU, a BrdU analog specifically incorporated in S-phase cells, and, after staining of both EdU and TSPYL2, we evaluated protein accumulation in those cells that after DNA damage progressed through S-phase (EdU positive). As shown in Supplementary Fig. 1F-G, no significant differences were found in TSPYL2 induction between EdU positive and negative cells, finally indicating that TSPYL2 accumulation is not restricted to S-phase.

Importantly, we observed that TSPYL2 accumulates only in female and Y-negative male cancer cells retaining high levels of γ-H2AX at late time points after DNA damage (Fig. 1H), and that, in response to prolonged etoposide treatment, it shows a granular staining with some spots co-localizing with DNA damage induced γ-H2AX foci (Supplementary Fig. 1H). This limited colocalization may be due to differences in the lesions or to the pan-nuclear TSPYL2 staining that masks its accumulation in foci. Nonetheless, these results indicate that TSPYL2 induction is promoted by treatment with genotoxic agents that cause persistent DSBs and that this protein may have a role in the response to this type of lesions.

### E2F1 promotes *TSPYL2* gene expression in response to DNA damage

**Fig. 2:**
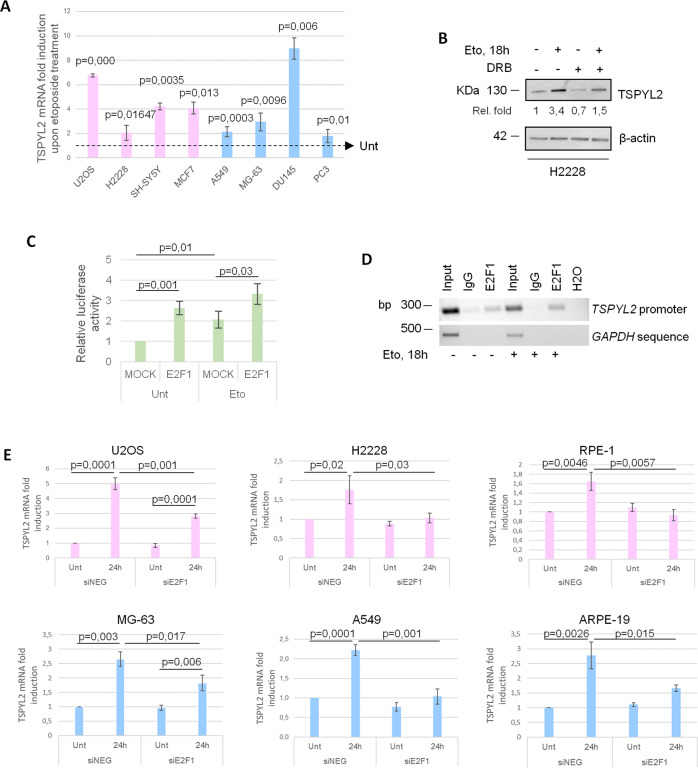
E2F1 promotes TSPYL2 gene expression in response to DNA damage. **A** RT-qPCR of TSPYL2 induction at 24 h after etoposide treatment in U2OS, H2228, SH-SY5Y, MCF7, A549, MG-63, DU145 and PC3 cell lines. P values were derived from Student’s t test between untreated and treated samples for each cell line. **B** Western blot analysis of TSPYL2 levels in H2228 cells treated with DRB (5,6-dichloro-1-beta-D-ribofuranosylbenzimidazole) and etoposide for 18 h in different combinations. **C** Luciferase assays of U2OS cells transfected with FLAG-E2F1 or empty vector (MOCK), before and after etoposide treatment for 6 h. P values were derived from Student’s t test between the indicated samples. **D** Chromatin immunoprecipitation (ChIP) assays of E2F1 on TSPYL2 promoter in untreated and etoposide-treated RPE-1 cells. Normal rabbit IgG were used as negative controls. Signals represent negative printing of ethidium bromide staining of PCR products. **E** RT-qPCR analysis of TSPYL2 mRNA levels before and after 24 h of etoposide treatment in control and E2F1 silenced U2OS, H2228, RPE-1, MG-63, A549 and ARPE-19 cell lines. P values were derived from Student’s t test between the indicated samples.

To investigate the mechanisms underlying TSPYL2 accumulation, we evaluated *TSPYL2* mRNA in different cancer cell lines before and after etoposide treatment. We found that, after DNA damage, *TSPYL2* gene expression is upregulated in all the tested cells, regardless of their sex (Fig. 2A) and that inhibition of gene transcription, by 5,6-dichloro-1-β-D-ribofuranosylbenzimidazole (DRB) treatment, reduces the etoposide-mediated increase of TSPYL2 protein levels (Fig. 2B).

These data demonstrate that after etoposide *TSPYL2* is induced also in those male cancer cells where the protein does not accumulate and that the increased gene transcription is necessary, but not sufficient, for TSPYL2 accumulation.

We next wanted to investigate the transcription factor responsible for *TSPYL2* upregulation upon genotoxic stress and we decided to investigate E2F1 involvement because of its role in the DDR [18] and in glioma sexual dimorphism [20, 21]. Using the ConTraV3 bioinformatic tool [27] and exploiting the MA0024.3 matrix of the JASPAR CORE database, we identified a putative E2F1 binding site conserved in many different vertebrate species on *TSPYL2* promoter (Supplementary Fig. 2A-B). To validate this prediction, we cloned the 500 bp promoter sequence of *TSPYL2* upstream of the luciferase reporter gene and we used this plasmid in combination with empty (MOCK) or FLAG-E2F1 encoding vectors to transfect U2OS cells. Luciferase assays demonstrated that *TSPYL2* promoter activity is significantly induced by etoposide treatment and further increased by ectopic E2F1 expression (Fig. 2C and Supplementary Fig. 2C).

We then evaluated E2F1 binding on *TSPYL2* promoter region by chromatin-immunoprecipitation (ChIP) and we found that E2F1 specifically associates with *TSPYL2* promoter in both unstressed and etoposide treated RPE-1 cells (Fig. 2D).

Finally, we silenced E2F1 in different normal and cancer cell lines of both sexes and no significant changes in *TSPYL2* mRNA levels were found in unstressed conditions (Fig. 2E). Conversely, after etoposide, E2F1 depletion strongly reduces or completely abolished the induction of *TSPYL2* expression (Fig. 2E).

Collectively, these results indicate that E2F1 binds TSPYL2 promoter before and after DNA damage and fosters *TSPYL2* gene transcription upon genotoxic stress.

### Genotoxic stress increases TSPYL2 protein stability in female but not in male cancer cells

As we found that TSPYL2 mRNA is upregulated in all the tested cell lines, we hypothesized that after DNA damage the protein may be degraded in male cancer cells. To verify this, we treated MG-63 and A549 cells with the proteasome inhibitor MG-132 and etoposide in different combinations and we examined TSPYL2 protein levels. As expected, we found that inhibition of protein degradation promotes TSPYL2 accumulation before and still more after etoposide (Supplementary Fig. 3).

**Fig. 3:**
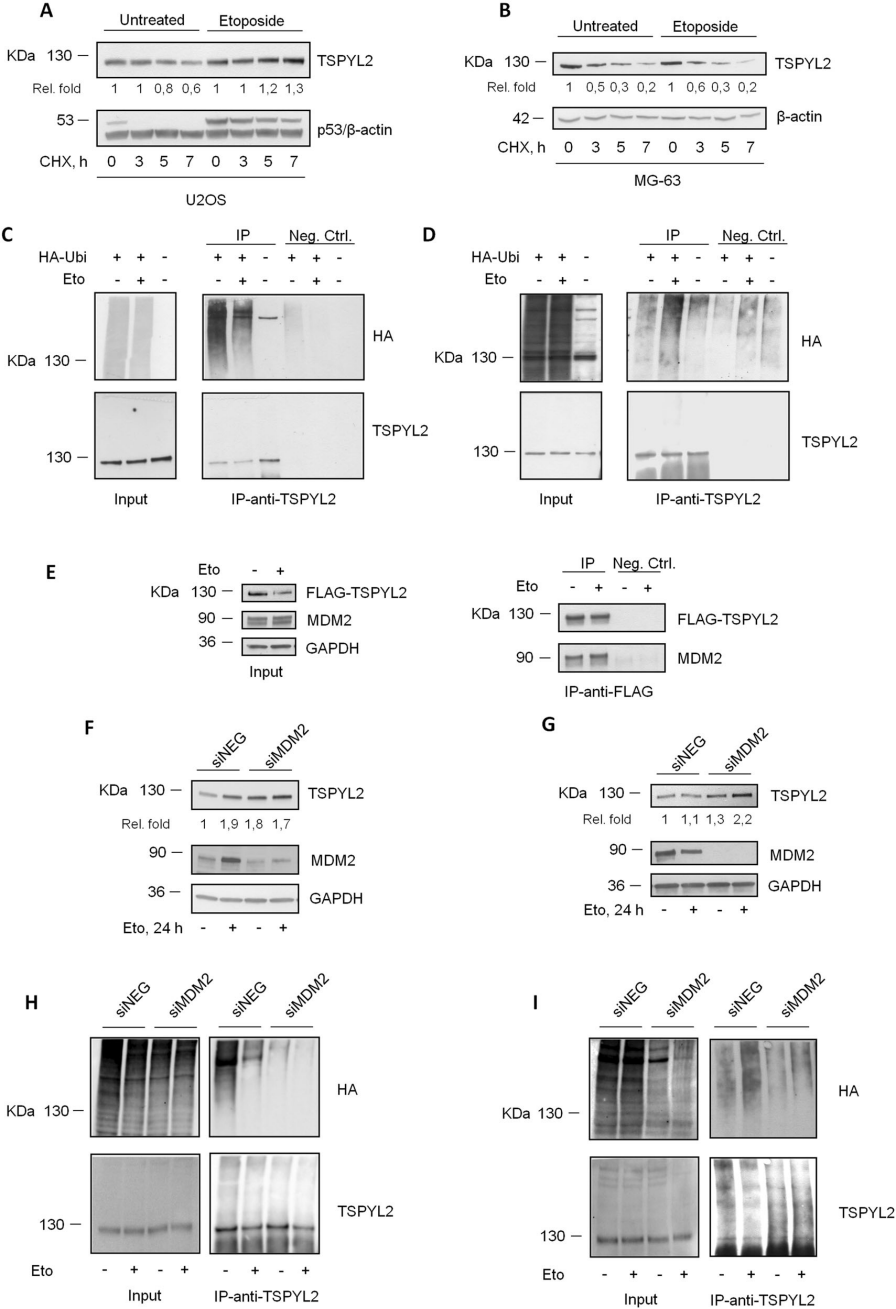
Genotoxic stress increases TSPYL2 protein stability in female but not in male cancer cells. **A** Western blot analysis of TSPYL2 levels in U2OS and **B** MG-63 cells treated with etoposide for 18 h and added with cycloheximide (CHX) for 3, 5, 7 h. **C** Western blot analysis of TSPYL2 immunoprecipitates from U2OS and **D** MG-63 cells transfected with HA-Ubiquitin plasmid, in untreated condition and after 8 h of etoposide treatment. **E** Western blot analysis of TSPYL2 immunoprecipitates from U2OS cells transfected with FLAG-TSPYL2 and MYC-MDM2, in untreated condition and after 24 h of etoposide treatment. **F** Western blot analysis of TSPYL2 induction at 24 h after etoposide treatment in MDM2 depleted U2OS and **G** MG-63 cells. **H** Western blot analysis of TSPYL2 immunoprecipitates from MDM2 depleted U2OS and **I** MG-63 cells transfected with HA-Ubiquitin plasmid, in untreated condition and after 8 h of etoposide treatment.

To extend these findings, we analyzed TSPYL2 protein stability in the osteosarcoma cell lines U2OS (female) and MG-63 (male). Cells were treated with the protein synthesis inhibitor cycloheximide (CHX) for different time points in the presence or absence of etoposide and we found that after DNA damage, TSPYL2 protein stability was increased in U2OS (Fig. 3A), and unaffected in MG-63 cells (Fig. 3B).

Then since proteasome-mediated degradation is generally regulated by ubiquitination, we transfected U2OS and MG-63 cells with a plasmid encoding HA-Ubiquitin and we immunoprecipitated TSPYL2 before and after DNA damage. We found that, in unstressed conditions, TSPYL2 is ubiquitinated, but, after DNA damage, this modification was reduced in the female U2OS (Fig. 3C) and increased in the male MG-63 cells (Fig. 3D).

Collectively these results suggest that, in unstressed conditions, TSPYL2 is regulated by ubiquitination and proteasome-dependent degradation, while after DNA damage, TSPYL2 is no longer ubiquitinated in female cancer cells but still targeted for degradation in male cancer cell lines.

The regulation of TSPYL2 in U2OS cells remembers that of p53, which is maintained at low levels by MDM2-dependent ubiquitination in the absence of genotoxic stress, but after DNA damage is quickly deubiquitinated and stabilized. Hence, to investigate if MDM2 has a role also in TSPYL2 stability, we initially analyzed their physical interaction in U2OS cells. Co-immunoprecipitation analyses revealed that ectopic FLAG-TSPYL2 and MYC-MDM2 associate both before and after DNA damage (Fig. 3E).

Then, transfecting U2OS with control or specific MDM2 siRNAs, we observed that MDM2 depletion increased TSPYL2 levels in unstressed conditions, but no further increment can be found after DNA damage (Fig. 3F). Differently, in MG-63 cells, MDM2 knock-down raised TSPYL2 levels before genotoxic stress and still more after etoposide treatment (Fig. 3G).

Finally, to confirm MDM2 role in TSPYL2 regulation, we analyzed TSPYL2 ubiquitination in MDM2-depleted cells and we found that loss of this ubiquitin-ligase reduces TSPYL2 ubiquitination in unstressed U2OS (Fig. 3H) and in both untreated and etoposide treated MG-63 (Fig. 3I).

These results therefore indicate that in unstressed conditions MDM2 regulates TSPYL2 protein levels in both U2OS and MG-63 cells, while after DNA damage it still ubiquitinates and degrades TSPYL2 only in the male MG-63 cell line.

### SRY represses TSPYL2 accumulation in male cancer cells after DNA damage

The occurrence of TSPYL2 induction in male cancer cells that lost the Y chromosome suggests that at least one factor implicated in the repression of TSPYL2 accumulation in males may be encoded by this chromosome.

**Fig. 4:**
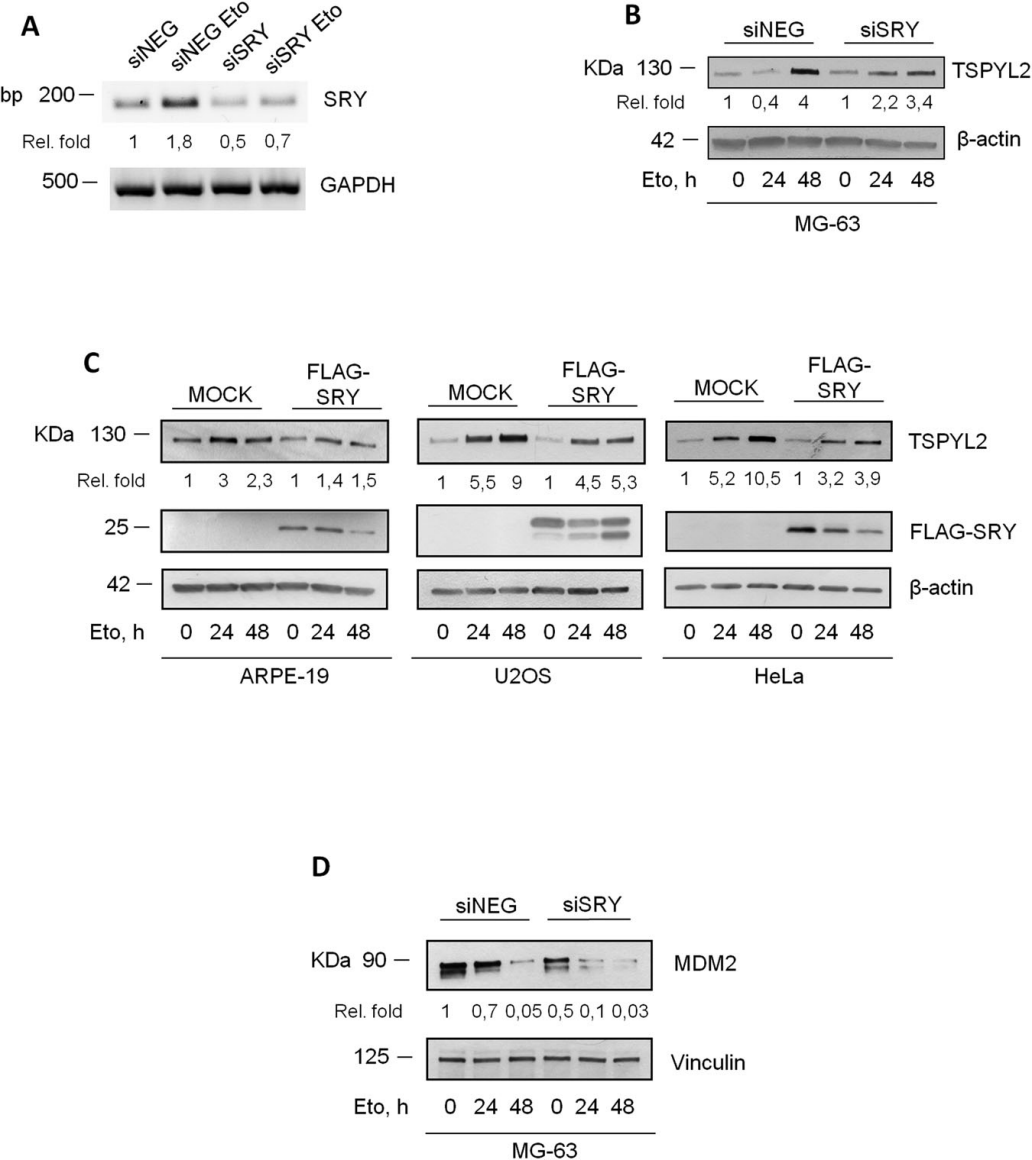
SRY represses TSPYL2 accumulation in male cancer cells after DNA damage. **A** Agarose gel electrophoresis of SRY RT-PCR products derived from untreated and etoposide-treated control and SRY silenced MG-63 cells. Signals represent negative printing of ethidium bromide staining. **B** Western blot analysis of TSPYL2 induction at 24 h and 48 h after etoposide treatment in SRY-depleted MG-63. **C** Western blot analysis of TSPYL2 induction at 24 h and 48 h after etoposide treatment in ARPE-19, U2OS and HeLa cells transfected with MOCK or SRY expression vector. **D** Western blot analysis of MDM2 levels at 24 h and 48 h after etoposide treatment in SRY-depleted MG-63 cells.

We initially examined the involvement of SRY [23, 24]. We showed that, in MG-63 cells, *SRY* gene is expressed, and its mRNA levels are induced after etoposide treatment (Fig. 4A), but, unfortunately, we could not find any specific antibody to verify SRY protein levels.

Nevertheless, we noticed that, in MG-63 cells, *SRY* depletion by siRNA transfection restores the pattern of TSPYL2 accumulation observed in normal and female cancer cells (Fig. 4B). Conversely, SRY overexpression in the female cancer cell lines U2OS and HeLa and in the male normal ARPE-19 cells is sufficient to reduce the accumulation of TSPYL2 at both 24 and 48 h after etoposide treatment (Fig. 4C). These results therefore indicate that, after DNA damage, SRY expression represses TSPYL2 induction in male cancer cells.

As we found that MDM2 is involved in TSPYL2 protein levels regulation, we investigated the possible interplay between SRY and MDM2 and we found that SRY silencing, in MG-63 cells, reduces MDM2 protein levels at both 24 and 48 h of etoposide treatment (Fig. 4D). These results therefore suggest that SRY may repress TSPYL2 accumulation by modulating MDM2 protein levels.

### TSPYL2 accumulation prevents cell growth in response to etoposide treatment

**Fig. 5:**
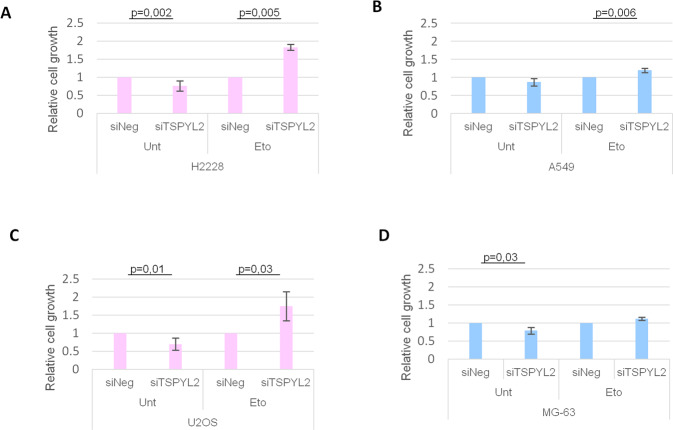
TSPYL2 regulates cell growth in response to DNA damage. Charts representing crystal violet assays in TSPYL2 silenced H2228 (**A**) and A549 (**B**) cells in untreated condition and after etoposide treatment. Graphical representation of cell growth assays in TSPYL2 silenced U2OS (**C**) and MG-63 (**D**) cells in unstressed condition and upon etoposide treatment. P values were derived from Student’s t test between untreated and treated samples for each cell line.

It was previously demonstrated that TSPYL2 overexpression arrests cellular proliferation [9, 28]. We therefore hypothesized that TSPYL2 accumulation may be useful to restrict cell growth upon DNA damage. To test this hypothesis, we silenced TSPYL2 in the lung cancer H2228 (female) and A549 (male) cell lines. Transfected cells were treated with 5 μM etoposide for 24 h and then their ability to proliferate and form colonies was evaluated. We found that TSPYL2 loss strongly increased the relative growth of H2228 cells in response to DNA damage (from 1 to 1.8, Fig. 5A), while only weakly increasing the fitness of A549 (from 1 to 1.2, Fig. 5B).

A similar experiment was performed with the osteosarcoma cell lines U2OS (female) and MG-63 (male). Control and TSPYL2-depleted cells were exposed to 20 μM etoposide for 24 h and then counted 24 h after release from drug treatment. We found that TSPYL2 depletion increased the relative growth of the female U2OS cells (from 1 to 1.75, Fig. 5C), but not that of the male MG-63 (from 1 to 1.1, Fig. 5D).

Importantly, these TSPYL2 mediated effects are not due to its role in apoptosis regulation because both U2OS (Supplementary Fig. 4A) and A549 (Supplementary Fig. 4B) cells depleted of TSPYL2 show reduced levels of p53 acetylation and apoptosis induction, as previously reported [12], but cellular proliferation after DNA damage was affected only in U2OS.

Altogether these results indicate that, after etoposide treatment, TSPYL2 loss promotes the proliferation of female cancer cells, where it accumulates, while leaving unaffected that of male cancer cells. Therefore, TSPYL2 accumulation is required to sustain cell growth arrest in response to DNA damage in female cancer cell lines.

### *TSPYL2* is more frequently mutated in female-specific cancers

**Fig. 6:**
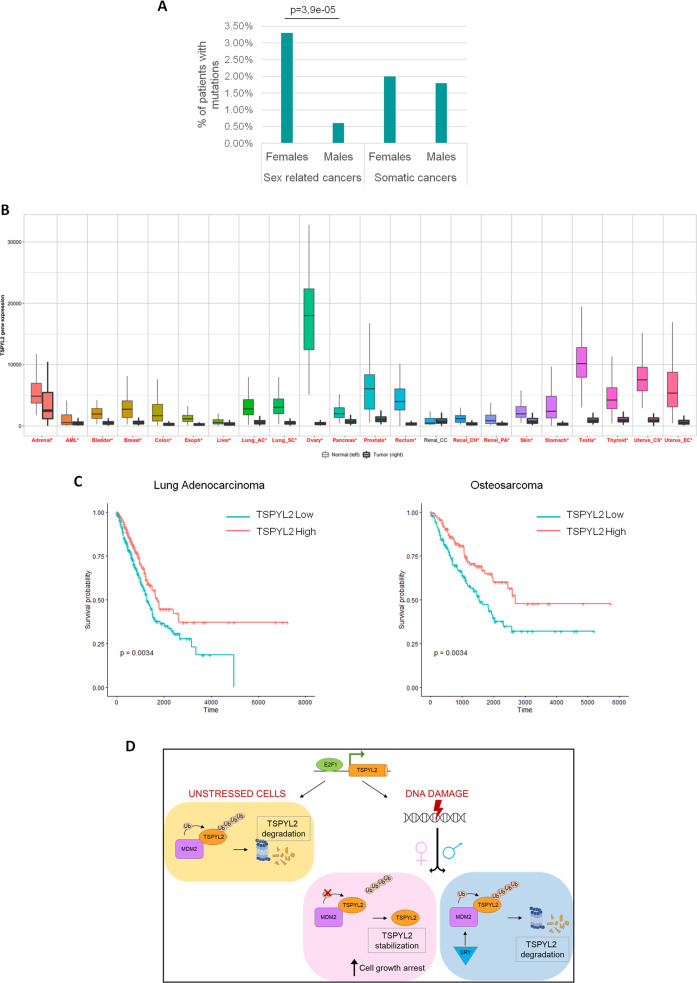
*TSPYL2* is more frequently mutated in female-specific cancers. **A** Distribution of mutations in TSPYL2 gene according to sex in sex-related and somatic cancers. Statistical significance was calculated using Fisher’s exact test. **B** TSPYL2 expression in tumor vs matched normal pairs according to TNMplot tool. **C** Kaplan-Meier plots showing the percentage of patients who are alive at a time point in lung adenocarcinoma and osteosarcoma according to TSPYL2 expression level (high in red and low in blue). Statistical significance was calculated using log-rank test. **D** Graphical scheme of our proposed model. In unstressed conditions, TSPYL2 is maintained at low levels by MDM2-dependent proteasome degradation. In response to DNA damage TSPYL2 protein is stabilized and accumulates in non-transformed and in female cancer cells, but not in male cancer cells where SRY expression promotes the MDM2-dependent proteasome degradation of TSPYL2. The accumulated protein promotes the arrest of damaged cell growth.

The similarity between TSPYL2 and p53 regulation and the finding that genes involved in the modulation of p53 function mutate more frequently in male tumors [29], prompted us to analyze *TSPYL2* status in human cancers. To this aim, we exploited The Cancer Genome Atlas (TCGA) database through the cBioPortal tool [30]. We found data about *TSPYL2* gene for 10953 cancer patients (Supplementary Fig. 5A), and we identified *TSPYL2* mutations in 225 of them (2%), with missense mutations being the most represented (37,3%), followed by amplifications (27,1%), homodeletions (22,7%) and truncating mutations (10,2%) (Supplementary Fig. 5B). However, when we compared the frequency of gene alterations between the sexes in the different tumors (Supplementary Table I), we observed that 84 out of 2540 (3,3%) female patients and 4 out of 655 (0,6%) males with sex-related tumors have mutations in *TSPYL2* gene (Fig. 6A). Differently, for somatic cancers, we found *TSPYL2* mutations in 59 out of 2906 (2%) females and 75 out of 4227 (1,8%) males (Fig. 6A). Therefore, these results indicate that *TSPYL2* is more frequently mutated in female-specific cancers, while no significant differences between sexes can be found in somatic tumors.

We also investigated mutations distribution, and we observed alterations scattered throughout TSPYL2 protein with hotspots falling inside the functional NAP and C-terminal domains for both female-specific and somatic tumors (Supplementary Fig. 5C). Even if it is not known if these mutations have loss of function effects on TSPYL2, these results suggest that this protein’s activities may have important roles in preventing somatic and female-specific cancer formation and progression. Unfortunately, no conclusions can be drawn for male-specific cancers because of the low number of identified mutations (2 missense, one amplification and one deletion).

Using the TNMplot web tool [31], we also compared *TSPYL2* expression level in different normal and tumor tissues (Fig. 6B) and we found that the mRNA of this gene is significantly reduced in almost all the investigated cancer specimens, further confirming a tumor suppressor role for TSPYL2, as previously suggested.

Finally, we analyzed patients’ survival in relation to *TSPYL2* expression levels in different tumors. Kaplan Meyer curves revealed that low expression of TSPYL2 well correlates with reduced survival in lung adenocarcinoma and osteosarcoma (Fig. 6C). On the contrary, no significant differences were found between female and male-specific cancers (Supplementary Fig. 5D). In addition, The Human protein Atlas reports that TSPYL2 expression can be prognostic for pancreatic, renal and colorectal cancer, with high expression being favorable in the former and unfavorable in the others (Supplementary Fig. 5E). Altogether these results suggest that TSPYL2 could have distinct functions in different tissues and tumors and that deregulation of its expression or mutations could variously impact on survival depending on the cancer type.

## Discussion

The DDR was until now considered to act equally in males and females and only recently, evidence of sex differences is emerging in these pathways [5].

Here we demonstrate, for the first time, that, after genotoxic stress, TSPYL2 protein accumulates in non-transformed cell lines. Differently, in cancer cells, TSPYL2 is regulated in a sex-specific manner and after DNA damage accumulates only in female cells. Furthermore, TSPYL2 induces a sex-dependent response to genotoxic stress in cancer cell lines, restricting cellular proliferation after DNA damage specifically in female cells. Our results therefore confirm the existence of sexual dimorphisms in the DDR and suggest that these disparities may contribute to the different chemotherapy outcomes in male and female patients.

We interestingly found that the differences in TSPYL2 regulation between males and females are due to the presence of the Y chromosome and more specifically to *SRY*, a gene that has no homologs in female cells [22]. *SRY* encodes for a transcription factor important for male determination during development but associated with oncogenic properties in adult tissues [23, 24]. We found that *SRY* transcription is induced after etoposide treatment and this finding, together with its role in TSPYL2 regulation after DNA damage, suggests for this protein a novel and sex-specific role in the DDR. Importantly, SRY is the first Y-linked gene whose function has been associated with the DDR.

Since loss of TSPYL2 accumulation in male cancer cells is not due to reduced transcription, we hypothesized that SRY may regulate TSPYL2 levels indirectly or through a transcription-independent mechanism. Accordingly, we demonstrated that SRY depletion in male cancer cells reduces the levels of MDM2, the ubiquitin ligase that we showed to regulate TSPYL2 protein turnover. However, it is also possible that, besides MDM2 and SRY, other proteins may be involved in the regulation of TSPYL2 stability before and after DNA damage. Indeed, TSPYL2 protein levels were previously reported to inversely correlate with UBE2C, another ubiquitin-conjugating enzyme [32], and most recently ZFP91, a zinc-finger protein, was found to mediate TSPYL2 destabilization [33]. In addition, also SRY is known to generally perform its function modulating the activity of SOX family members, whose role in TSPYL2 regulation has not been yet investigated.

The MDM2-dependent regulation of TSPYL2 in female cancer cells suggest for this protein an important role in the regulation of cellular proliferation, just like p53 [34]. Since it was previously reported that TSPYL2 controls cell cycle progression inhibiting cyclin B1-CDK1 complex [9], it is conceivable that its levels should be finely regulated to prevent the arrest of cell proliferation in the absence of DNA damage. On the contrary, TSPYL2 accumulation after genotoxic stress may contribute to prevent the proliferation of damaged cells and preserve genome stability. Accordingly, we found that, after etoposide, TSPYL2 is required to restrict cellular proliferation specifically in female cancer cells, where the protein is induced by DNA damage, suggesting the importance of TSPYL2 upregulation in these events. These TSPYL2-dependent effects do not rely on its role in apoptosis regulation because both the p53 wild-type cell lines U2OS and A549 depleted of TSPYL2 show defects in apoptosis induction, but only in the female cells TSPYL2 regulates cellular proliferation after DNA damage. However, considering the timing of TSPYL2 accumulation after DNA damage and its partial co-localization with DNA damage induced γH2AX foci, we cannot exclude for this protein other functions in the late events of the DDR. In fact, in consideration of our previously published data [12] and of our new findings, we hypothesize a dual role for TSPYL2 in the DDR. Initially TSPYL2, by regulating p53 acetylation, is involved in the first steps of the DDR to promote p53 function and apoptosis induction. Then TSPYL2 is induced to participate to the late events of the DDR, and in particular to restrict the proliferation of cells exposed to genotoxic agents that cause persistent DSBs. Importantly, at least in cancer cells, this TSPYL2 function is sex-specific since expression of the male-specific gene SRY prevents TSPYL2 accumulation in male cancer cells. However, we cannot exclude other sex-related or unrelated functions for TSPYL2 in the DDR. For example, the E2F1-mediated transcriptional regulation of TSPYL2 may suggest its involvement in E2F1-dependent cell cycle regulation or apoptosis induction. Moreover, its ability to regulate SIRT1 activity may suggest a role in the regulation of DNA repair.

However, as a further proof of TSPYL2 role in the restriction of damaged cell growth, we demonstrated that the transcription factor responsible for *TSPYL2* upregulation after genotoxic stress is E2F1, a well-known regulator of genes implicated in cell cycle control, DNA replication, DNA repair and apoptosis [18]. Indeed, E2F1 silencing completely abrogates the induction of *TSPYL2* expression in response to etoposide in different normal and cancer male and female cell lines. Instead, in U2OS and MG-63 cells, the absence of E2F1 only reduces *TSPYL2* expression, therefore indicating that in these cell lines other transcription factor(s) could be involved in the regulation of this gene. Nevertheless, it is also possible that other mechanisms, modulating, for example, mRNA stability, contribute to the fine tuning of *TSPYL2* expression in these cells.

Exploiting the TCGA database to find TSPYL2 mutations in human cancers, we unexpectedly found that this gene is more frequently mutated in female-specific cancers and that gene modifications mostly affect the functional domains of the protein. Despite this, no significant association between the reduced *TSPYL2* expression and survival could be observed in sex-specific tumors, while a correlation was found in lung adenocarcinoma, osteosarcoma and pancreatic cancer. This is in contrast with recent reports demonstrating that X-linked genes involved in p53 regulation are more frequently mutated in males [29] and suggests that TSPYL2 could also have important functions in other cellular pathways implicated in cancer prevention. Accordingly, we found that *TSPYL2* expression is significantly reduced in almost all the analyzed cancer tissues, confirming its tumor suppressor role. On the contrary, we also found in The Human Protein Atlas database that in renal and colorectal cancer, TSPYL2 expression is associated with unfavorable prognosis, therefore suggesting for this protein tissues and tumors specific functions.

Collectively, as depicted in Fig. 6D, our results suggest a model in which, in unstressed conditions, TSPYL2 is maintained at low levels by MDM2-dependent degradation, but after DNA damage, in normal and female cancer cells, it is stabilized through E2F1-dependent gene transcription and protein deubiquitination. The accumulated TSPYL2 then prevents the proliferation of damaged cells. These events may instead be defective in male cancer cells, where in response to genotoxic stress TSPYL2 expression is still induced by E2F1, but the protein undergoes MDM2-dependent ubiquitination and degradation. These results could therefore contribute to explain the highest predisposition to cancer and the worst prognosis of men compared to women.

Although we cannot exclude other relevant functions for the accumulated TSPYL2, these findings importantly suggest that in the future this protein may be a promising target for cancer therapy since its sex-specific regulation could provide novel insights and opportunities for the development of personalized cancer therapy. In fact, the proper modulation of TSPYL2 expression in both male and female patients may regulate p53 function and cell cycle arrest, finally increasing the sensitivity of patients to chemotherapy.

## Materials and Methods

### Cell culture

All cell lines were obtained from the American Type Culture Collection (ATCC) or from the European Collection of Authenticated Cell Cultures (ECACC) and periodically tested for mycoplasma contamination. The human cancer cell lines U2OS, A549, DU145, PC-3, SH-SY5Y, HT-1080, SW480, HCT-116, HeLa, MCF-7 and the human normal cell lines MRC-5-STR-V were cultured in DMEM (Lonza) supplemented with 10% fetal bovine serum (FBS), 5000 U/ml penicillin and 5 mg/ml streptomycin; the human cancer cells H2228, SKOV3, Jurkat and MDA-MB-231 were grown in RPMI (Lonza) supplemented with 10% FBS, 5000 U/ml penicillin and 5 mg/ml streptomycin; human primary fibroblasts, MG-63, RPE-1 and ARPE-19 cell lines were maintained in MEM (Lonza) supplemented with 10 % FBS, 5000 U/ml penicillin and 5 mg/ml streptomycin. The human nontumorigenic mammary epithelial cell line HME was cultured in DMEM/F12 supplemented with 10% FBS, insulin (5 μg/ml), hydrocortisone (1 μg/ml) and human epidermal growth factor (10 ng/ml). All cell lines were maintained at 37 °C and 5% CO2. 10 μg/ml Hygromycin B was added to RPE-1 cells.

### Cells transfections and treatments

Plasmid and siRNAs transfections were carried out using Lipofectamine 2000 and RNAiMAX (Thermo Fisher Scientific), respectively, according to the manufacturers’ instructions. siRNAs against TSPYL2 (cat. S34364 and S34364) were purchased from Ambion, siNEG (cat. 1027281), siMDM2 (cat. SI00300846) and siE2F1 (cat. SI00073976 and SI00073990) from QIAGEN, siSRY (cat. M-011780-00-0005) and siP53 (cat. L-003329-00-0005) were SMARTpool from Dharmacon.

Unless differently indicated, DNA damage was induced by treating cells with etoposide (20 μM Merck), NCS (8,8 nM, Merck), UV (20 J/m2), gemcitabine (10 μM, Merck), hydroxyurea (1 mM), camptothecin (20 μM, Merck) and nocodazole (80 ng/ml, Merck) for different time points. DRB (Merck) was used concentrated at 20 μM. MG132 (Merck) was added 20 min before etoposide treatment, at the concentration of 5 μM, unless otherwise indicated. CHX (Sigma) was used at 10 μg/ml.

### Immunofluorescence

Immunofluorescence stainings were performed essentially as described [35]. U2OS cells grown on coverslips were treated with 20 μM etoposide for the indicated time points. Then, cells were fixed with 4% paraformaldehyde, permeabilized with 0.5% Triton X-100 in PBS, blocked in 3% BSA in PBS, stained with TSPYL2 (Bethyl), or with TSPYL2 and γ-H2AX (Merck) antibodies and DAPI. Images were acquired using Zeiss AxioImager M2 microscope (40X NA 1.3). Mean intensity was automatically calculated using a custom CellProfiler 4.1 pipeline. For S-phase analyses, EdU was added with etoposide for 24 h or for 15 min to untreated cells. Click-iT-EdU Alexa Fluor 488 (Thermofisher) staining was performed as previously described [35] and followed by an immunostaining step to reveal TSPYL2 using an Alexa-Fluor 555 conjugated anti-rabbit as secondary antibody. Experiments were repeated three times and the percentage of cells with accumulated TSPYL2 in EdU positive and negative cells was determined by two independent operators.

### Western blot and antibodies

Western blot analyses were performed on total cell extracts using the NuPAGE system (Thermo Fisher Scientific) or the Mini PROTEAN TGX gels and the Trans-Blot Turbo Transfer System (Biorad). Antibodies used were: p53-DO7 (Santa Cruz Bio-technology, cat. Sc-47698), TSPYL2 (Bethyl, cat. A304-013A), E2F1, MDM2, cleaved PARP-1 and p53-Ac-K382 (Cell Signalling Technology, respectively cat. 3742 S, 86934 S, 5625 S and 2525 S), γ-H2AX-JBW301, β-actin, GAPDH, FLAG and HA (12CA5) (Merck, respectively cat. 05-6363, A1978, SAB1405848, F1804 and 11583816001). Densitometric analyses were performed with the Fiji software (Schindelin et al. 2012). Original and uncropped western blot images can be found in Supplementary material.

### RT-qPCR and PCR

Total RNA was extracted using the RNeasy Mini Kit (QIAGEN), according to manufacturer’s instructions and quantified using NanoPhotometer P330 (Implen). 1 μg of total RNA was retro-transcribed using the SuperScript IV First-Strand Synthesis System (Thermo Fisher Scientific). qPCR was performed in triplicate on 20 ng of cDNA using QuantiFast SYBR Green PCR Kit (Qiagen) and the LightCycler 480 System (Roche). Reactions were performed in 20 μl of final volume in triplicates. Samples were normalized using GAPDH as reference gene. Experiments were repeated three times. Primer sequences were: TSPYL2_for AGGCACTGGAGGATATTCAG; TSPYL2_rev GAAGGGTCTTCGCATCTGGAT; GAPDH_for ACCACAGTCCATGCCATCAC; GAPDH-rev TCCACCACCCTGTTGCTGTA. PCR analyses were performed with the GoTaq G2 Flexi DNA Polymerase kit (Promega) according to manufacturer’s procedure and using the following conditions: 95 °C for 5 minutes, 35 cycles of PCR (95 °C for 30 seconds, 64 °C for 30 seconds, 72 °C for 30 seconds) and 72 °C for 10 minutes. The amplification products were loaded on 2% agarose gels. Primers used were: SRY_for GCATTCATCGTGTGGTCTCG; SRY_rev TTCGCTGCAGAGTACCGAAG; GAPDH primers were the same used for qPCR.

### Luciferase assays

The 500 bp upstream of the *TSPYL2* gene start site were cloned in the pGL4.11[luc2P] vector (Promega) to drive the expression of the luciferase reporter gene luc2P (Photinus pyralis). This plasmid was then used to transfect U2OS cells together with pRL-TK (encoding Renilla luciferase, Promega) and FLAG-E2F1 (GenScript) plasmids. After 48 h, cells were exposed to 20 μM etoposide for 6 h or left untreated and luciferase activity was analyzed with the Dual-Luciferase Reporter Assay System (Promega) and normalized over Renilla luciferase activity according to manufacturer protocol. Experiments were repeated three times and the mean and standard deviation were reported in the chart.

### Immunoprecipitation

Immunoprecipitations were performed essentially as previously described [36]. Briefly, for ubiquitination analysis, U2OS and MG63 cells were transfected with MOCK or HA-Ubiquitin vectors and, two days after transfection, cells were treated with 10 μM MG132 and 20 μM etoposide for 8 h before harvesting. Cells were lysed in ELB lysis buffer (150 mM NaCl, 50 mM Hepes pH 7.5, 5 mM EDTA, 0.5% NP-40) supplemented with protease inhibitors cocktail and 10 mM N-ethyl maleimide (NEM, Merck) and lysates were precleared for 30 min with protein A coupled sepharose resin (Sigma). Precleared lysates were then incubated with specific anti-TSPYL2 antibody and immunoprecipitations were carried on for 3 h. After washes, immunocomplexes were detached from the resins with Laemmli buffer and analyzed by western blot.

For co-immunoprecipitation assays, U2OS cells were transfected with FLAG-TSPYL2 [12] and Myc3-Mdm2 (Addgene 20935; [37] encoding vectors and treated or not with 20 μM etoposide for 16 h. Cells were then lysed in ELB buffer supplemented with protease inhibitors cocktail and, after preclearing with protein A coupled Sepharose resin, immunoprecipitations were carried on with anti-FLAG antibody for 3 h. Samples were then washed and analyzed by western blot as described above.

### Flow cytometry analyses

Flow cytometry analyses to evaluate DNA content were performed as previously described [35]. Cells were fixed in 70% ethanol and then stained with 50 μg/ml propidium iodide in PBS-0.1% Tween supplemented with 5 μg/ml of RNaseA. Samples were then analyzed using an S3 flow cytometer (Biorad, Hercules, CA, USA) and FlowJo Software (BD Biosciences).

### Chromatin immunoprecipitation (ChIP)

RPE-1 cells were treated with 10 μM MG132 and 20 μM etoposide for 18 h. Cells were cross-linked with 1% formaldehyde and the chromatin was sheared into 500 bp DNA fragments by sonication with Bioruptor Sonicator (Diagenode). Fragmented DNA was incubated overnight with anti-IgG (1:100) or anti-E2F1 (Cell Signalling, 1:100) antibodies and then bound to Dynabeads protein-G (Thermo-Fisher). After extensive washing, IP samples and Input were incubated overnight with RNAse A at 65 °C and then treated with Proteinase K at 50 °C for 3 h. Chromatin was then purified with Chromatin IP DNA Purification Kit (Active Motif). E2F1 binding to *TSPYL2* promoter was determined through PCR using the following conditions: 95 °C for 5 minutes, 35 cycles of PCR (95 °C for 30 seconds, 62 °C for 30 seconds, 72 °C for 30 seconds) and 72 °C for 10 minutes. Primer sequences were: forward GGAGCCAATCGGAAACTGAT; reverse CTACTCCCTCCGCGCCAATC. Samples were normalized using GAPDH as reference gene.

### Crystal violet assay

H2228 and A549 cells were transfected with control or TSPYL2 siRNAs, treated with 5 μM etoposide for 24 h and seeded in triplicates in 6 wells plates. Two weeks later, cells were stained with crystal violet solution (0,2% crystal violet, 2% EtOH, H_2_O) and plates were washed in tap water twice. Then, stained cells were incubated with 1% SDS and quantified at 570 nm absorbance. Experiments were repeated at least three times and mean and standard deviation were reported in the charts.

### Cell growth analysis

Cell growth analysis were previously described [38]. Briefly, U2OS and MG-63 cells were transfected with control or TSPYL2 siRNAs and 24 h later 20,000 cells were seeded in triplicates in 6 wells plates. The next day cells were left untreated or exposed to 20 μM etoposide for 24 h and then released in drug-free medium. Grown cells were counted 24 h after release and the ratio between etoposide-treated and untreated cells was determined and reported in the chart. Experiments were repeated ≥3 times.

### Mutations and expression data

The PanCancer dataset (*n* = 10953 patients) from The Cancer Genome Atlas (TCGA) was investigated using the cBioPortal (http://www.cbioportal.org) tool [30]. Genetic alteration in TSPYL2 gene, such as mutation (in-frame, missense, splice, truncating), amplification and homodeletion were retrieved for different cancer types. Data were analysed considering the tumor type, sex, and survival status of the patients with or without TSPYL2 genetic alterations. The expression level of TSPYL2 in different tumors and matched normal pairs were plotted and compared using TNMplot (https://tnmplot.com/analysis/) [31]. Comparisons were performed using the Mann-Whitney U test with a statistical significance threshold set at *P* Value < 0.05.

### Survival plot

The survival analysis was performed using Cox model to determine the effect of gene expression levels on patients’ survival. Kaplan-Meier plot and log-rank test were used to observe the differences in gene expression status. The cut-off value for TSPYL2 expression was defined using the median expression value. The gene expression status for each patient was defined accordingly to the cut-off. The difference between the two groups (high/low expression) was statistically assessed by log-rank test (*P* value < 0.05 was considered significant).

The survival analysis was performed using the following R packages combined by a custom R script: the *UCSCXenaTools* package [39] was used for the data retrieval from TCGA; the *survival* (https://cran.r-project.org/web/packages/survival/index.html) and *survminer* (https://cran.r-project.org/web/packages/survminer/index.html) packages were used to create models and plot survival curves, respectively. The R version used is 4.1.2.

### Statistical analysis

Statistical significance of the differences between two groups mean values was determined using Student’s t test. The statistical significance of the difference between TSPYL2 mutation frequency in males and females was assessed using Fisher’s exact test. *P* value < 0.05 was considered significant. For survival plot, the difference between high and low expression groups was statistically assessed by log-rank test (*P* value < 0.05 was considered significant). Comparison of the growth of different cell lines was performed with ANOVA test using Prism 9 (GraphPad). All the experiments were performed at least twice, unless otherwise stated, and representative results are shown.

## Supplementary information

Supplementary Material

Uncropped western blot

Reproducibility checklist

## Data Availability

All the data are available upon request.
