# Supplementary material for: Sex specific regulation of TSPY-Like 2 in the DNA damage response of cancer cells
Source: Cell Death Dis. 2023 Mar 15;14(3):197. doi: 10.1038/s41419-023-05722-2 (PMC10015022; doi:10.1038/s41419-023-05722-2)
Supplement: Supplementary file 1 — Supplementary Material [file 41419_2023_5722_MOESM1_ESM.pdf]

SUPPLEMENTARY FIGURE LEGENDS:

Supplementary Fig. 1. A) Western blot analysis of cleaved-PARP-1, p53 and γ-H2AX protein levels in U2OS cells in untreated condition and after 1, 3, 6, 18 and 24 hrs of etoposide. **B)** Scatter plot shows the mean intensity of TSPYL2 in U2OS nuclei in unstressed condition and after etoposide treatment. Average ± S.E.M are indicated in red. P-value was calculated using Student's t-test. **C)** Proliferation rate analyses of the indicated cancer cell lines. No statistically significant difference among the cell lines was found according to ANOVA test. **D)** Cell cycle profile analyses by flow cytometry of the indicated cancer cell lines. **E)** Western blot analysis of TSPYL2 levels in different human cancer cell lines in untreated conditions. **F)** Examples of EdU and TSPYL2 staining in untreated and etoposide treated cells. **G)** Chart representing the percentage of EdU positive and negative cells with accumulated TSPYL2 after etoposide treatment. **H)** Immunofluorescence staining of TSPYL2 and γ-H2AX in untreated and etoposide treated U2OS cells.

Supplementary Fig. 2. A) The MA0024.3 matrix of the JASPAR CORE database (left) and visualization of E2F1 binding site position between -164bp and -176bp from the TSPYL2 ATG start site (right). **B)** E2F1 binding site on TSPYL2 promoter is conserved among species. **C)** Western blot analysis of ectopic E2F1 levels upon etoposide treatment in U2OS cells transfected with MOCK and FLAG-E2F1 expression vectors, used for luciferase assays.

Supplementary Fig. 3. Western blot analysis of TSPYL2 levels in MG-63 (left) and A549 (right) cells treated with MG-132 for 8 hrs, in untreated and etoposide-treated conditions.

Supplementary Fig. 4. A) Western blot analysis of cleaved PARP-1 and acetylated-p53 levels after etoposide treatment for 24 and 48 hrs, in TSPYL2 depleted U2OS and **B)** MG-63 cells.

Supplementary Fig. 5. A) TCGA data available for TSPYL2 gene according to the sex in different cancer types. **B)** Types and distribution of TSPYL2 mutations in the different cancer cases of the TCGA database. **C)** Mutations localization along TSPYL2 protein in all, sex-related and somatic tumors. **D)** Kaplan-Meier plots showing the percentage of patients who are alive at a time point in different sex specific tumors according to TSPYL2 expression level (high in red and low in blue). **E)** Kaplan-Meier plots, obtained from "The Human Protein Atlas" web site, summarizing the prognostic role of TSPYL2.

Supplementary Table I

List of the tumor types investigated during TCGA data analyses aimed at the identification of TSPYL2 mutations.

Supplementary Figure 1

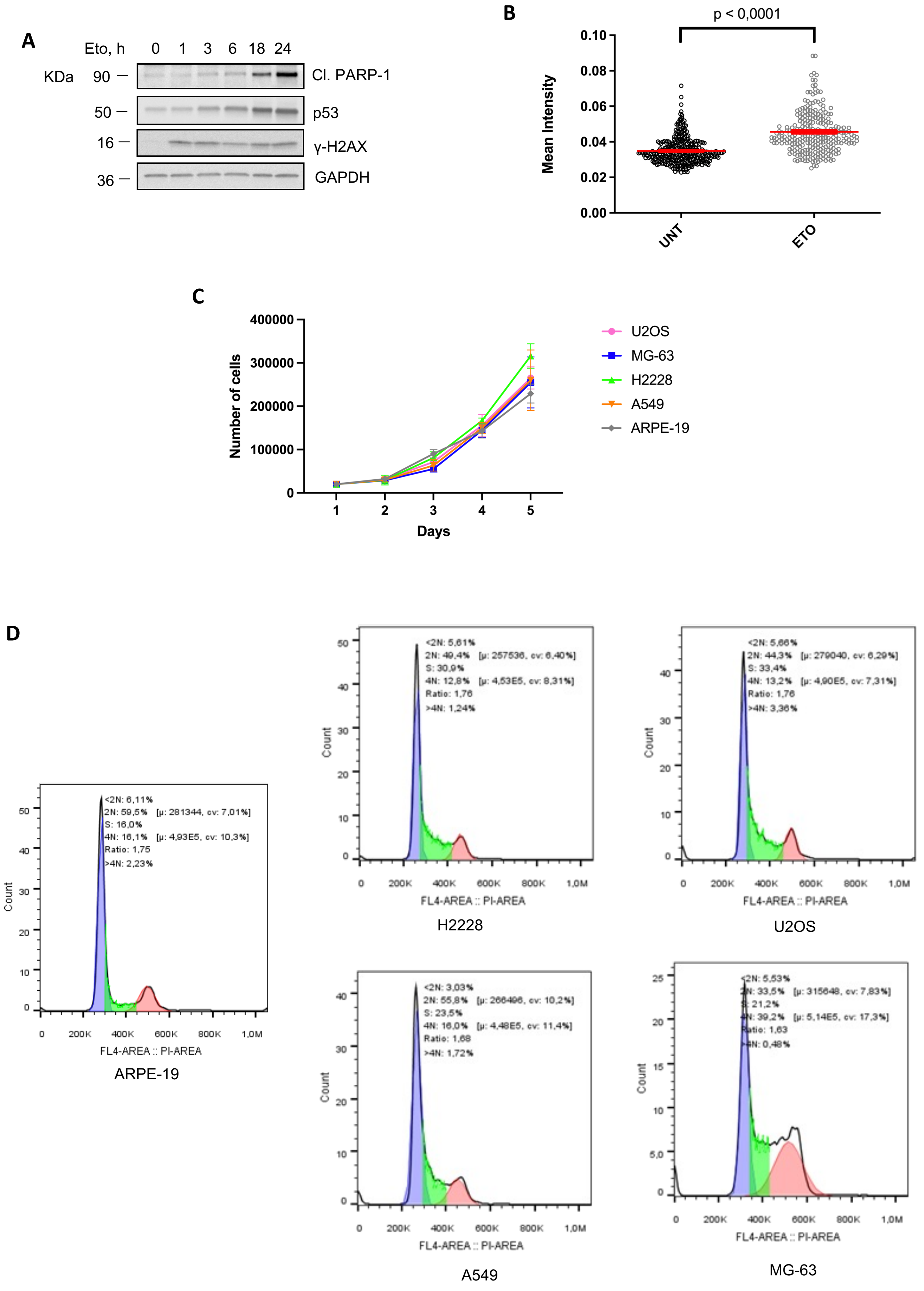

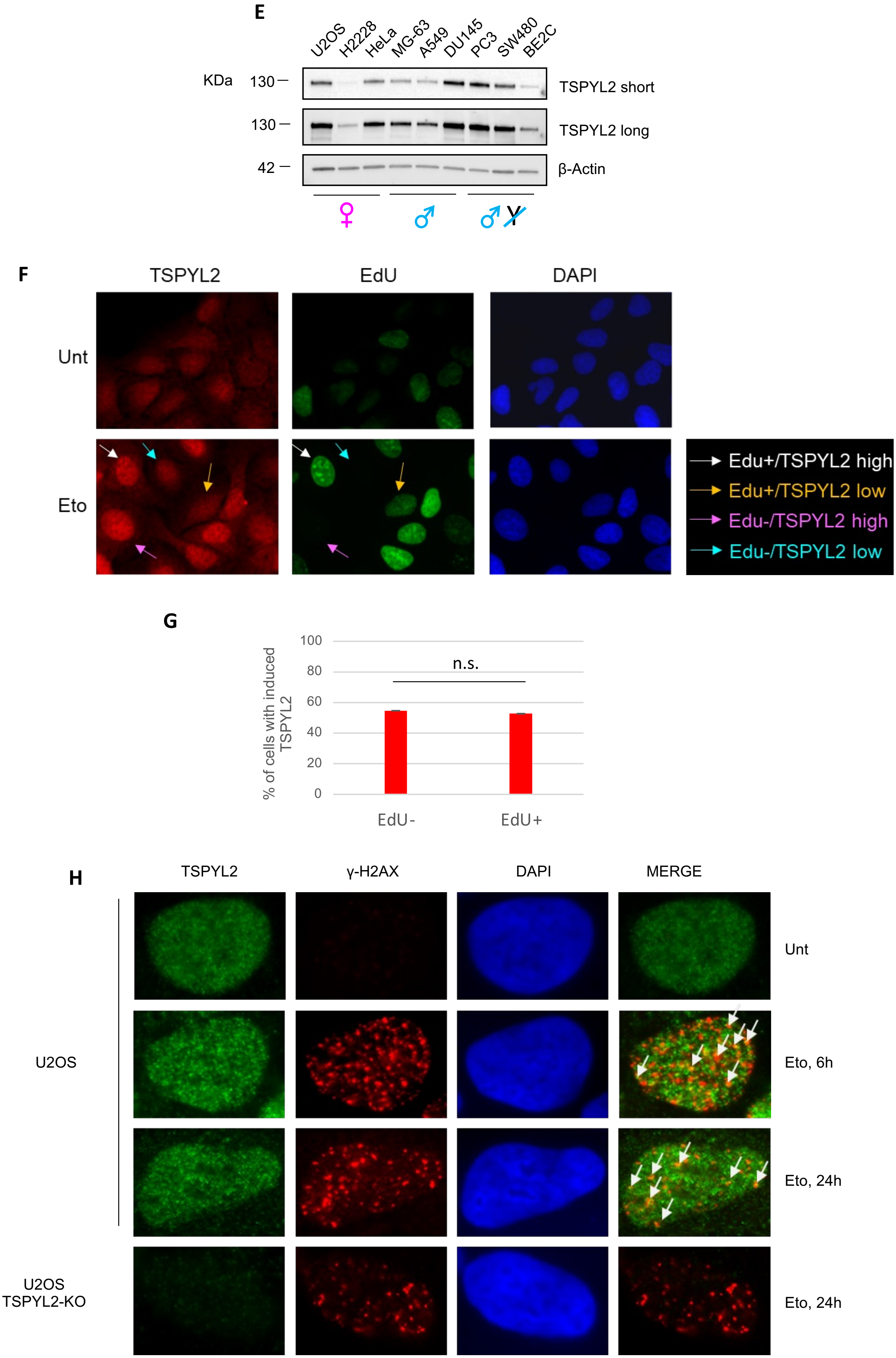

E
U2OS
H2228
HeLa
MG-63
A549
DU145
PC3
SW480
BE2C
KDa
130
130
42
TSPYL2 short
TSPYL2 long
β-Actin
F
TSPYL2
EdU
DAPI
Unt
Eto
Edu+/TSPYL2 high
Edu+/TSPYL2 low
Edu-/TSPYL2 high
Edu-/TSPYL2 low
G
% of cells with induced TSPYL2
100
80
60
40
20
0
n.s.
EdU-
EdU+
H
TSPYL2
γ-H2AX
DAPI
MERGE
U2OS
U2OS TSPYL2-KO
Unt
Eto, 6h
Eto, 24h
Eto, 24h

Supplementary Figure 2

A

E2F1 JASPAR_CORE_2016,MA0024.3,MA0024.3

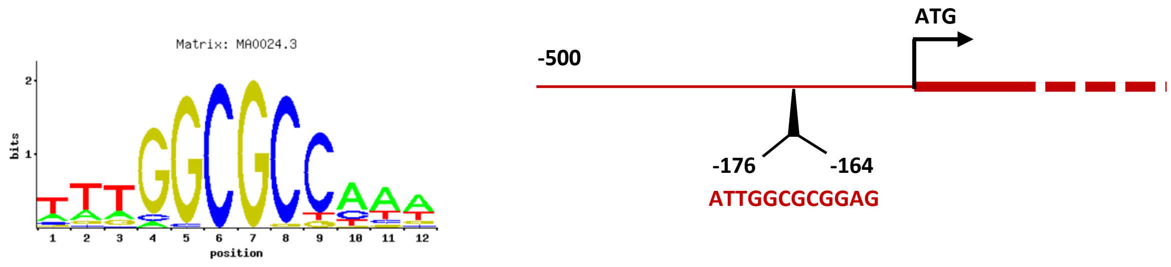

B

human/1-35	1	aggagtgggcggggcttcggcga-ttggcgcgg-ag-g	35
orangutan/1-35	1	aggagtgggcggggcctcggcga-ttggcgcgg-ag-g	35
rhesus/1-35	1	aggagcgggcggggcctcggcga-ttggcgcgg-ag-g	35
baboon/1-35	1	aggagcgggcggggcctcggcga-ttggcgcgg-ag-g	35
marmoset/1-35	1	aggaggggcggggcttcagcga-ttggcgcgg-ag-g	35
bushbaby/1-30	1	agaag-gggccgggctttggcta-----agcca-ca-g	30
treeshrew/1-36	1	aggagtgggccggggcttggcgg-attgcgcgg-agag	36
mouse/1-35	1	gggagtgggcggggcctgggcga-taggcgctg-tg-g	35
guinea-pig/1-35	1	aagagtgggcggggctttggcaa-ttggcgcgg-ag-g	35
kangaroo-rat/1-35	1	aggagtgggcgaggcttcagagg-ctggcgcgg-ag-g	35
pika/1-35	1	acgagtgggcgggatctctacga-ttggcgcgg-ag-g	35
rabbit/1-35	1	aggagtgggcgggacctcgacga-ttggcgcgg-ag-g	35
alpaca/1-33	1	-gcaggggcggtgcca-ggcgc-ttggcgcgg-ag-g	33
cow/1-33	1	-tgagggggcggggcct-gacga-ttggcgcgg-ag-g	33
dolphin/1-34	1	gcgaggggcggggtct-ggcgc-ttggcgcgg-ag-g	34
cat/1-35	1	cggagagggcggggccttgacgc-ttggcgcgg-ag-g	35
dog/1-35	1	gggaggggcgggtcctgggcgc-ttggcgcgg-ag-g	35
horse/1-33	1	gggagagggaggg--ccgggcgc-ttggcgcgg-ag-g	33
microbat/1-34	1	gcg-aggggcgggacccgagcag-ttggcgcgg-ag-g	34
megabat/1-31	1	gggaagggacgggacctgggc---tcggcgcg---g-g	31
hedgehog/1-35	1	gaggaagggagggccggcgcct-ttggcgcgg-ag-g	35
elephant/1-35	1	gggtgggggcgggacattggcgg-ttggcgcgg-ag-g	35
rock-hyrax/1-35	1	gggaggggcggaacgttggcgg-ttggcgcgg-ag-g	35
armadillo/1-34	1	---agagggcgggaaccttgcggattggcgcggaag-g	34

C

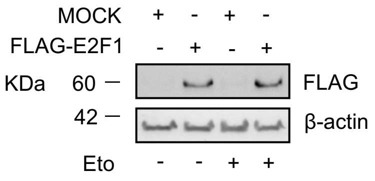

Supplementary Figure 3

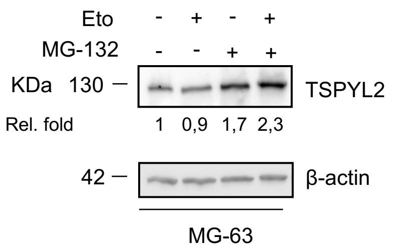

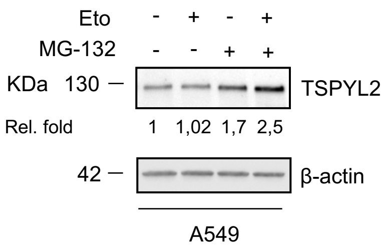

Supplementary Figure 4

A

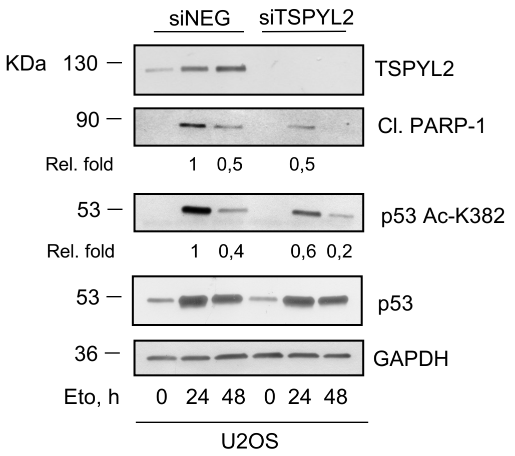

B

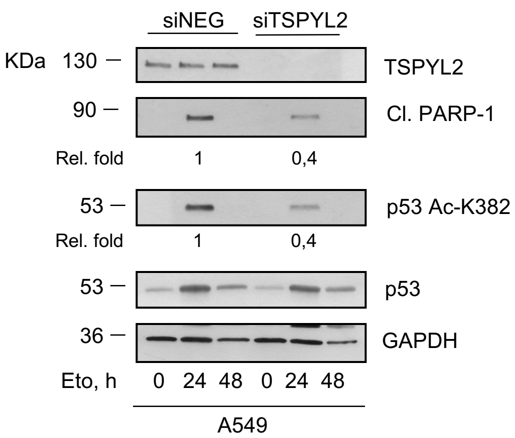

Supplementary Figure 5

A

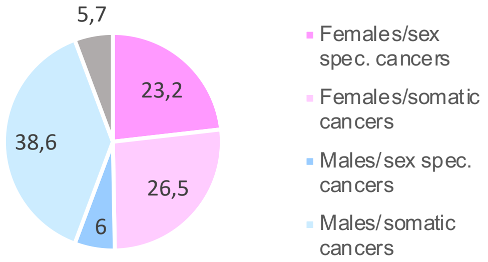

B

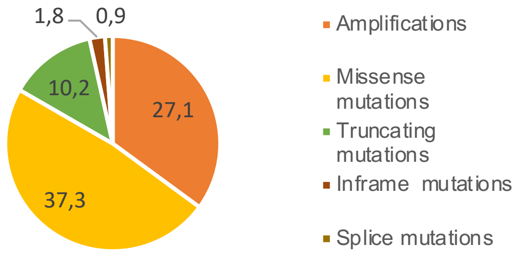

C

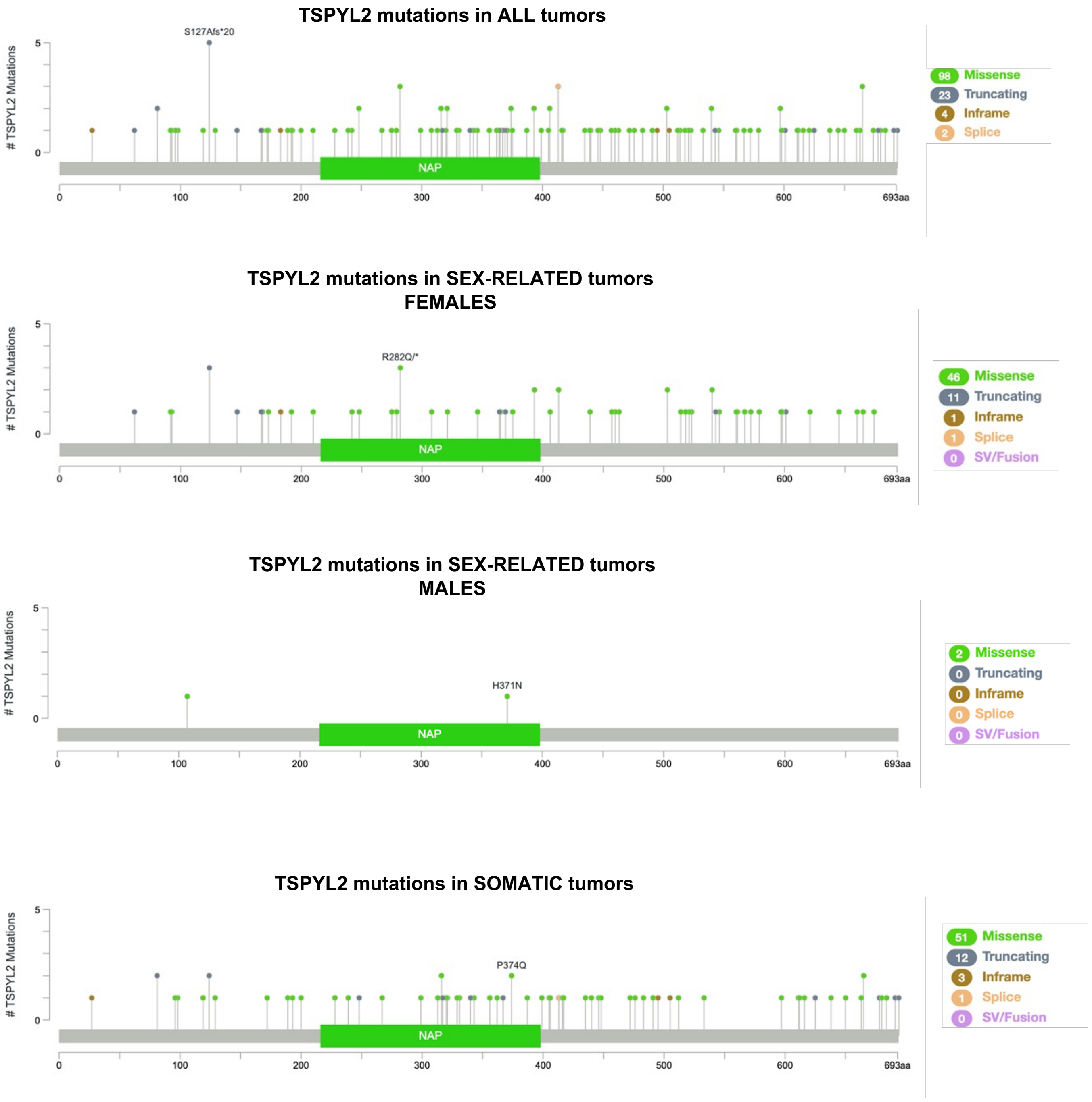

D

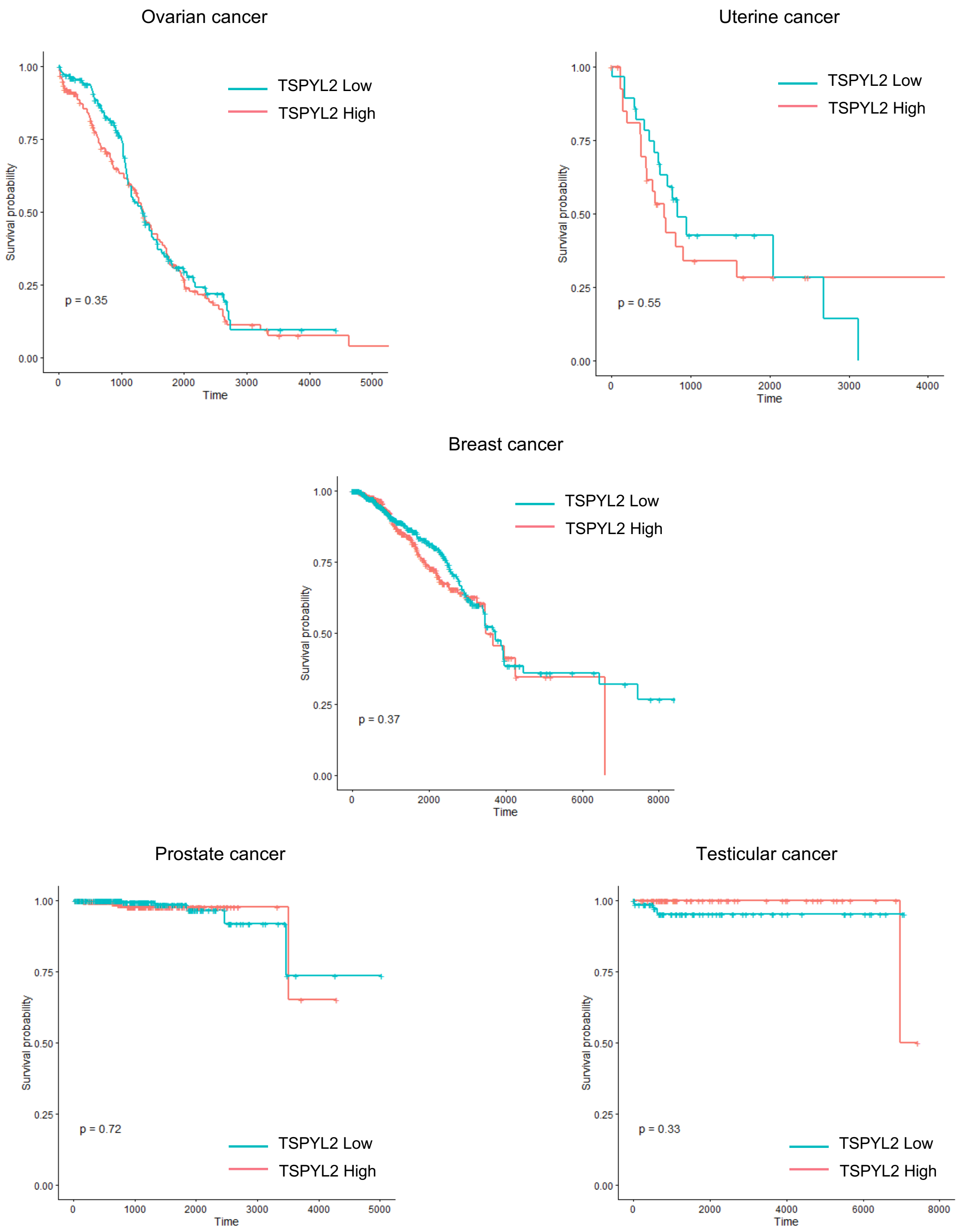

Ovarian cancer
TSPYL2 Low
TSPYL2 High
p = 0.35
Survival probability
Time
Uterine cancer
TSPYL2 Low
TSPYL2 High
p = 0.55
Survival probability
Time
Breast cancer
TSPYL2 Low
TSPYL2 High
p = 0.37
Survival probability
Time
Prostate cancer
p = 0.72
TSPYL2 Low
TSPYL2 High
Survival probability
Time
Testicular cancer
p = 0.33
TSPYL2 Low
TSPYL2 High
Survival probability
Time

E

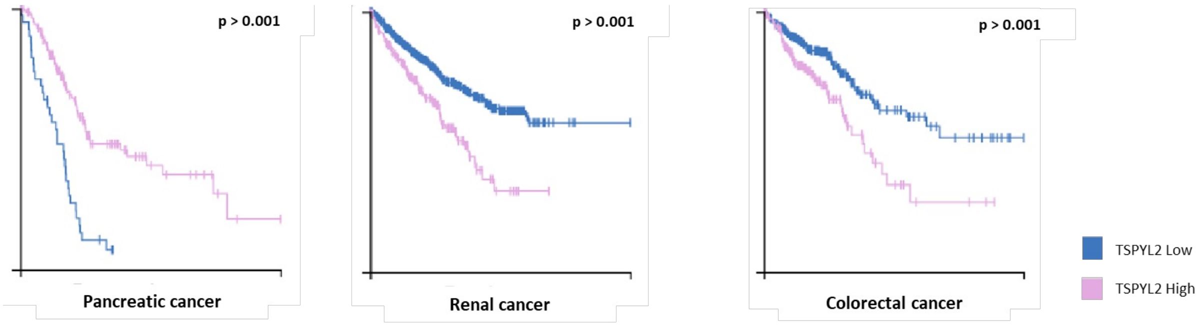

p > 0.001
Pancreatic cancer
p > 0.001
Renal cancer
p > 0.001
Colorectal cancer
TSPYL2 Low
TSPYL2 High

Supplementary Table I

SEX-RELATED TUMORS	
**FEMALE**	**MALE**
Cervical Squamous Cell Carcinoma	Prostate Adenocarcinoma
Ovarian Serous Cystadenocarcinoma	Testicular Germ Cell Tumors
Uterine Carcinoma	
Breast Invasive Carcinoma	

SOMATIC TUMORS
Acute Myeloid Leukemia
Adrenocortical Carcinoma
Bladder Urothelial Carcinoma
Brain Lower Grade Glioma
Cholangiocarcinoma
Colorectal Adenocarcinoma
Diffuse Large B-Cell Lymphoma
Esophageal Carcinoma
Glioblastoma Multiforme
Head and Neck Squamous Cell Carcinoma
Kidney Carcinoma
Liver Carcinoma
Lung Carcinoma
Mesothelioma
Pancreatic Adenocarcinoma
Pheocromocytoma and Paraganglioma
Sarcoma
Melanoma
Stomach Adenocarcinoma
Thymoma
Thyroid Carcinoma
