# Supplementary material for: Sex specific regulation of TSPY-Like 2 in the DNA damage response of cancer cells
Source: Cell Death Dis. 2023 Mar 15;14(3):197. doi: 10.1038/s41419-023-05722-2 (PMC10015022; doi:10.1038/s41419-023-05722-2)

Fig. 1B

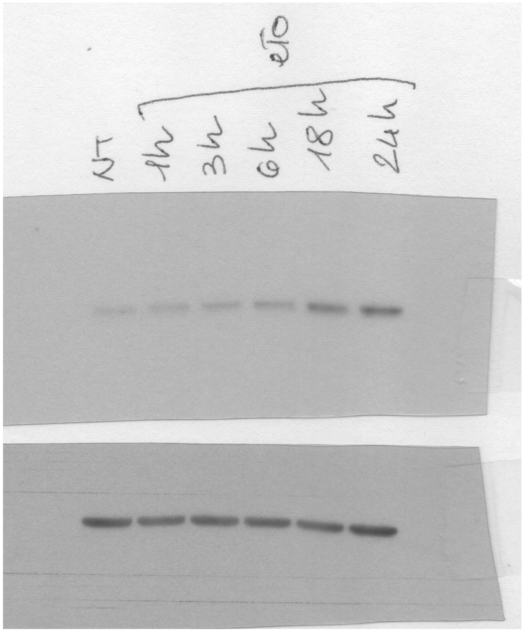

TSPYL2

β-actin

Fig. 1D top

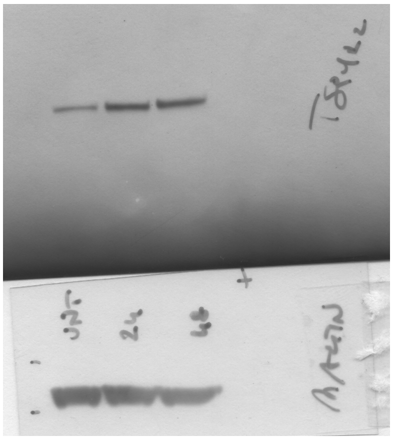

TSPYL2

β-actin

Fibroblasts

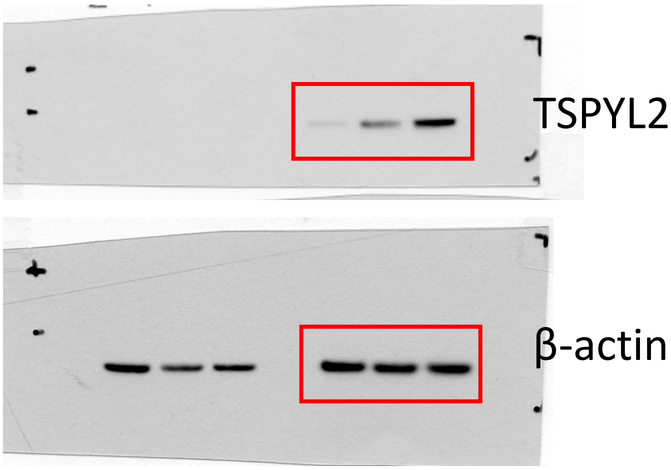

TSPYL2

β-actin

HME-1

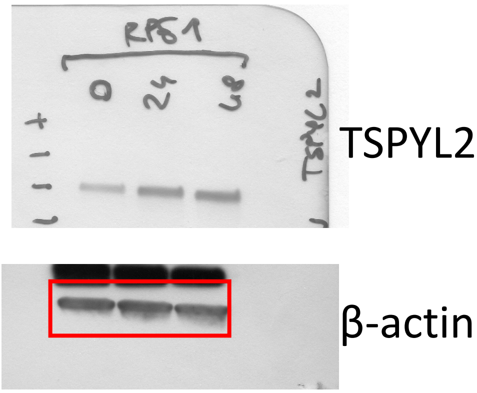

TSPYL2

β-actin

RPE-1

Fig. 1D bottom

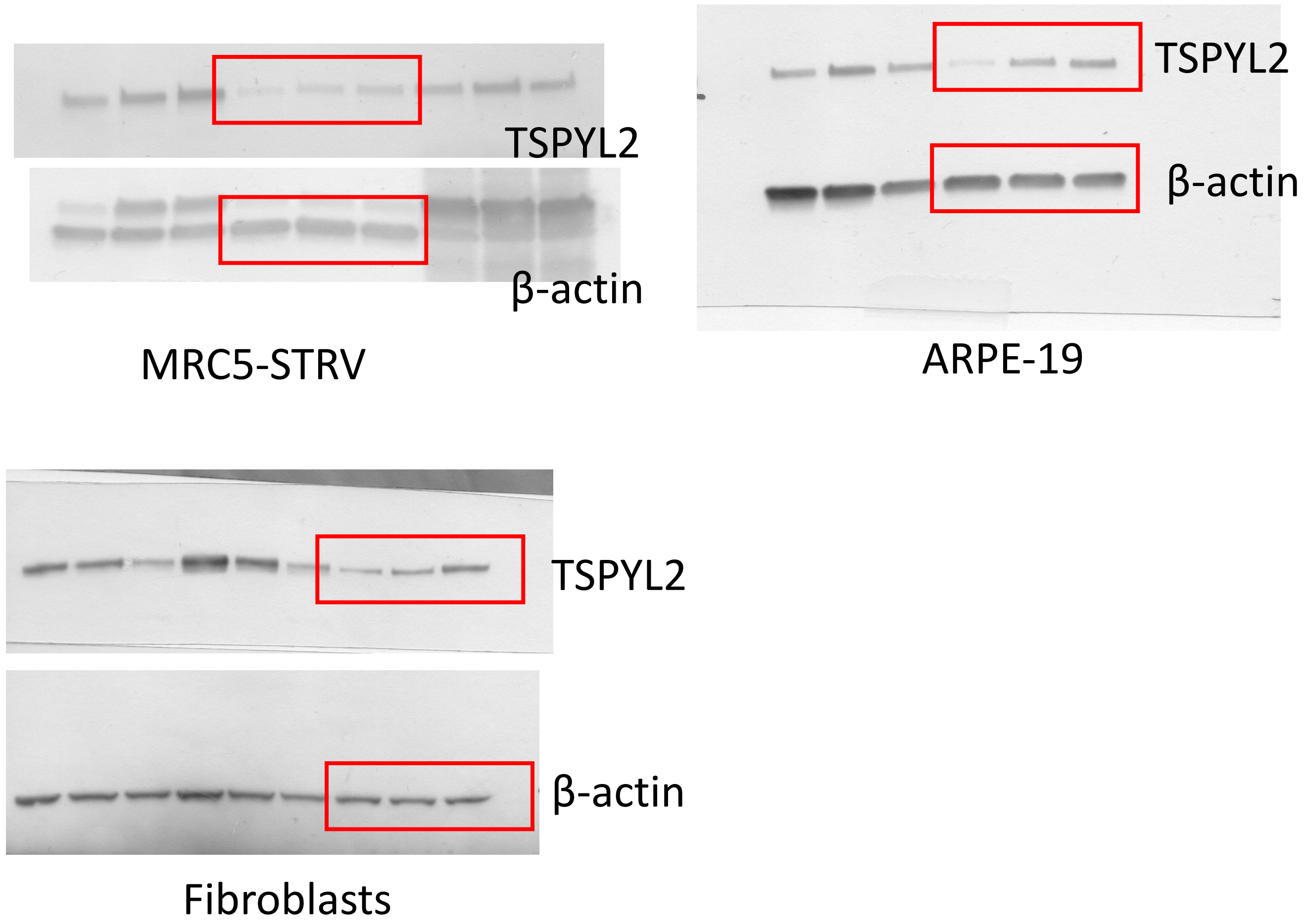

Fig. 1E

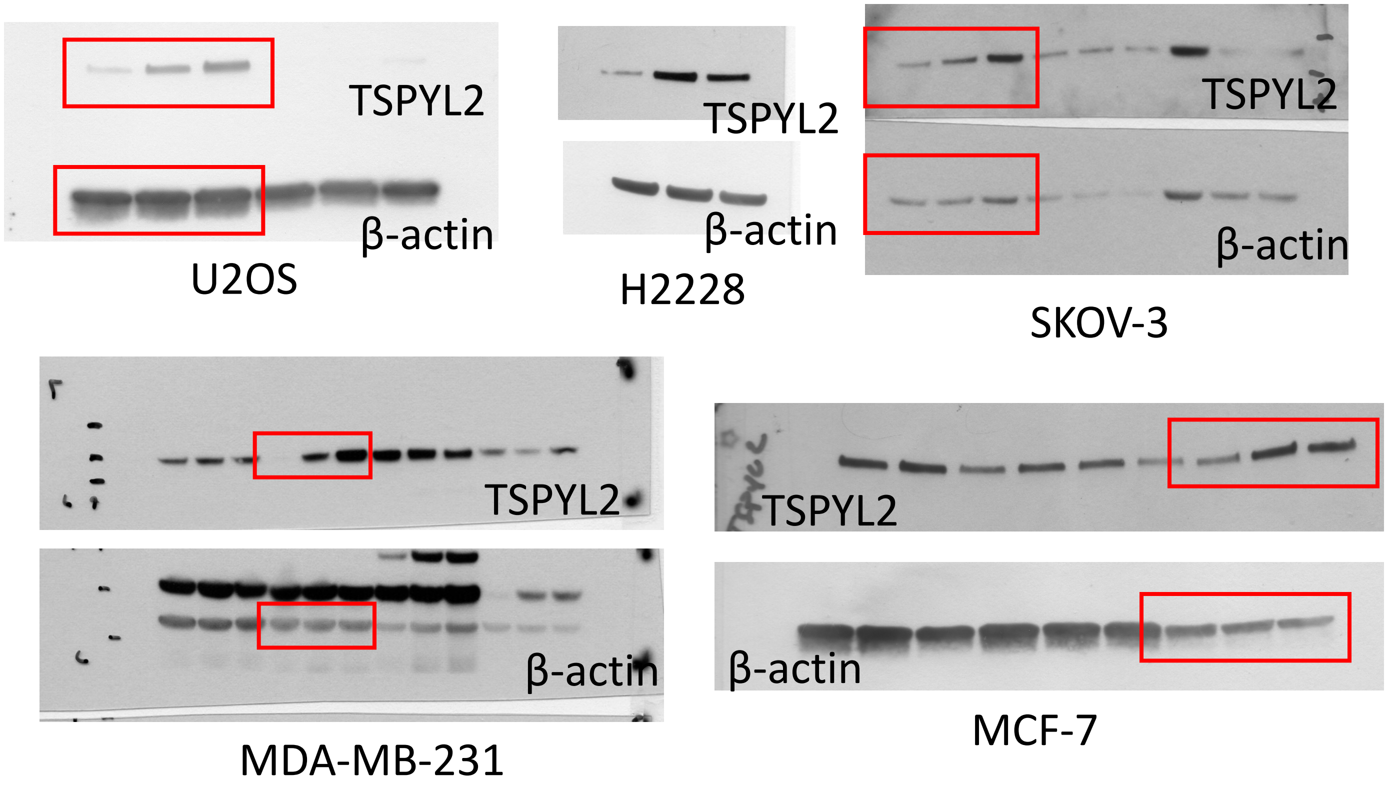

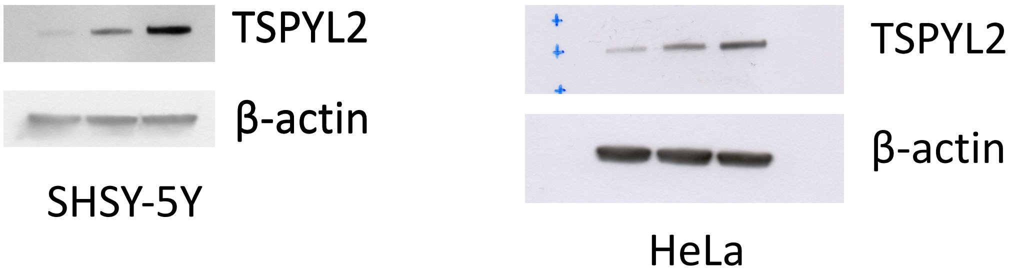
TSPYL2
β-actin
SHSY-5Y
TSPYL2
β-actin
HeLa

Fig. 1F

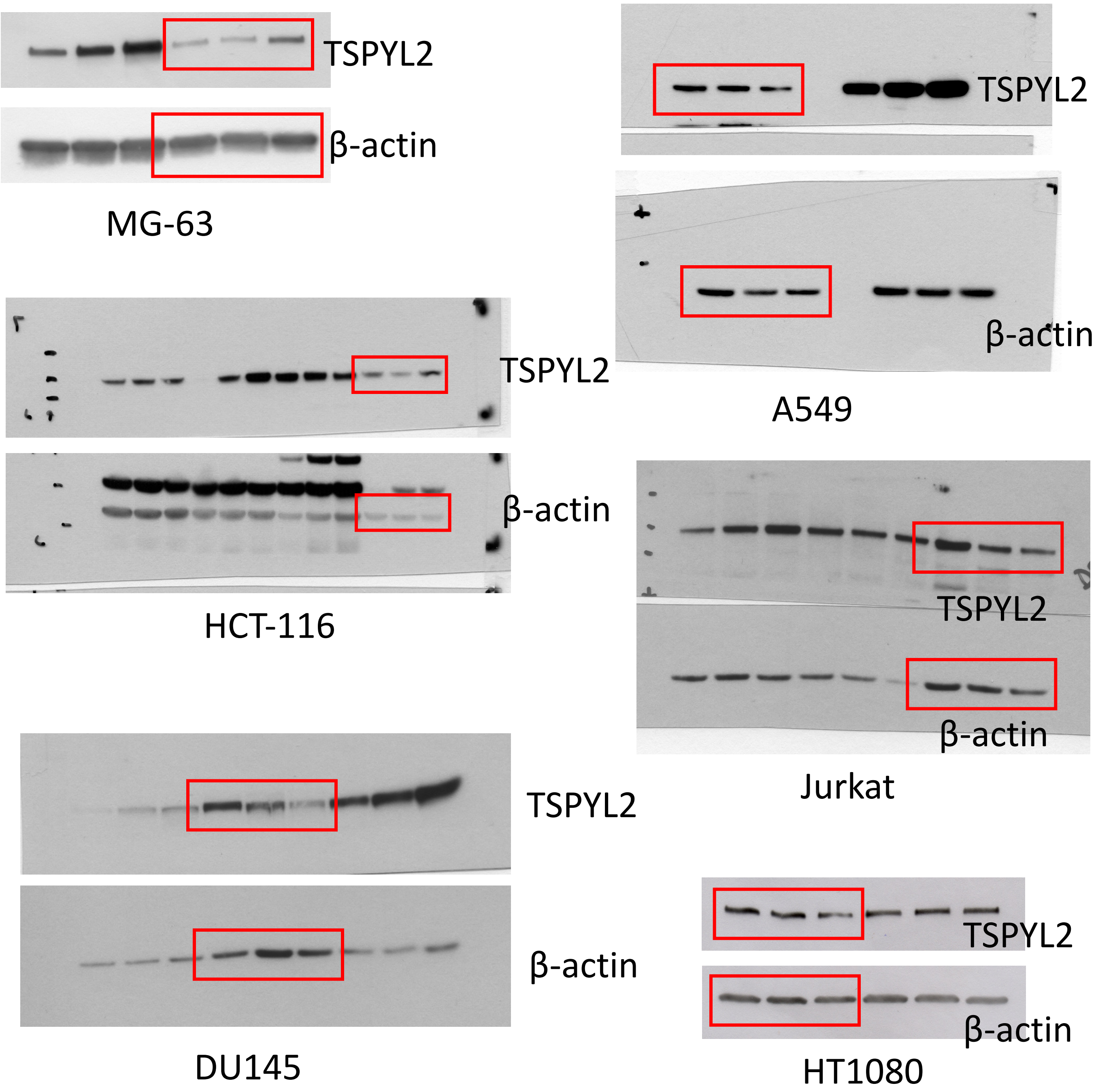
TSPYL2
β-actin
MG-63
TSPYL2
β-actin
A549
TSPYL2
β-actin
HCT-116
TSPYL2
β-actin
Jurkat
TSPYL2
β-actin
DU145
TSPYL2
β-actin
HT1080

Fig. 1G

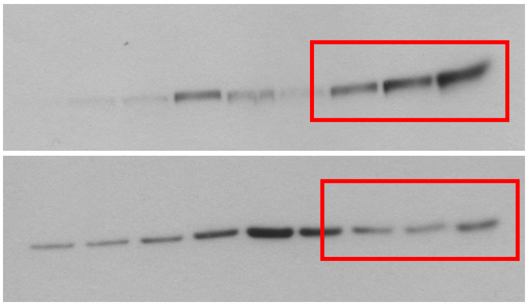

TSPYL2

β-actin

PC-3

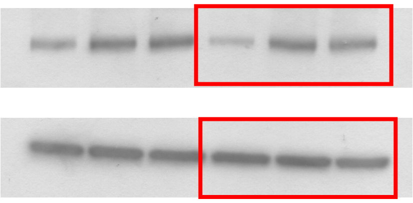

TSPYL2

β-actin

SW480

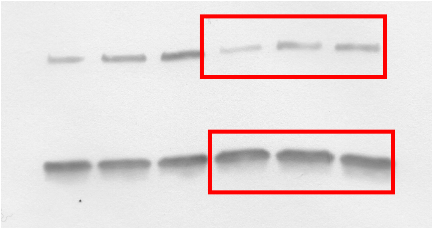

TSPYL2

β-actin

BE(2C)

Fig. 1H

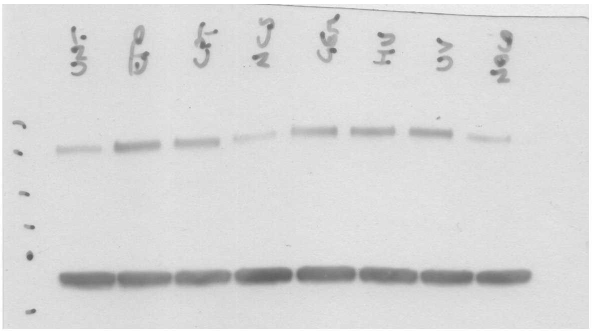

TSPYL2

β-actin

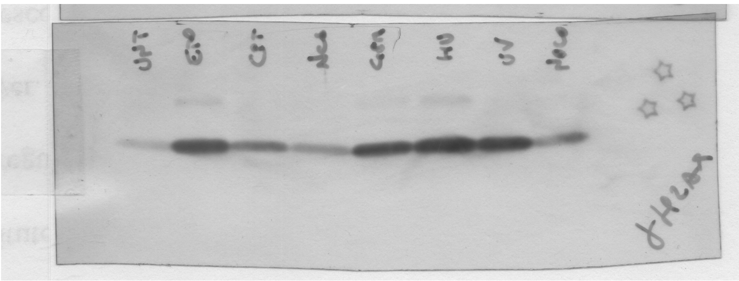

γ-H2AX

U2OS

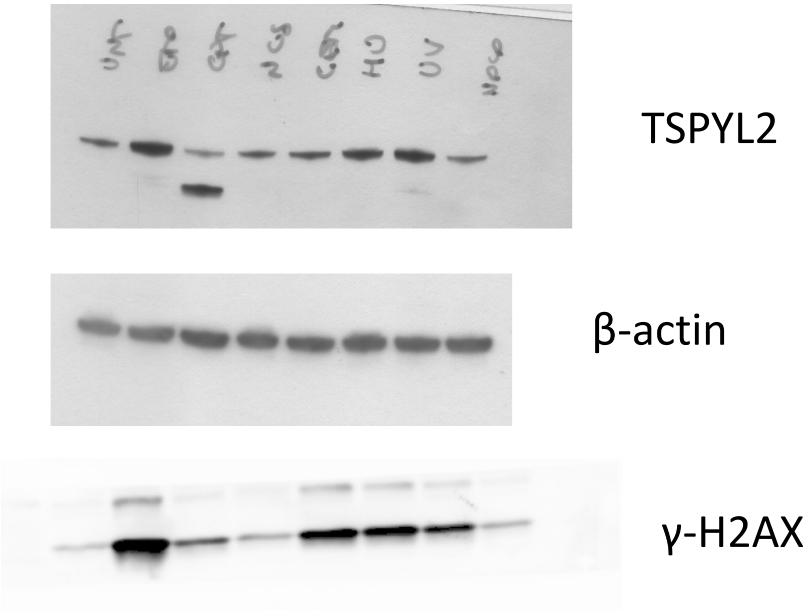

SW480

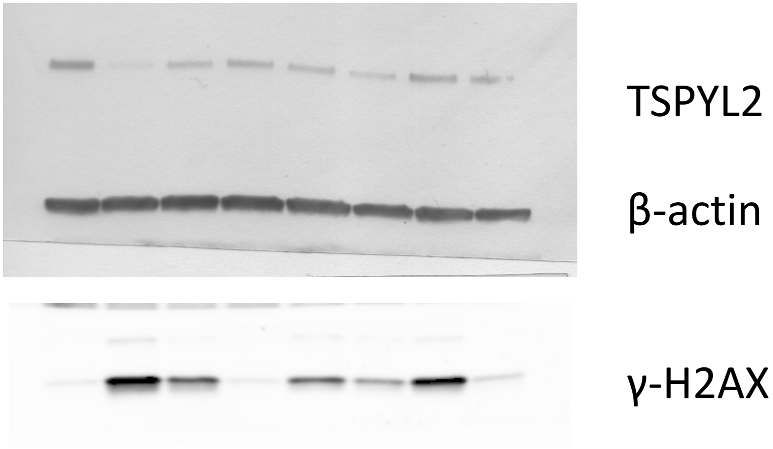

DU145

Fig. 2B

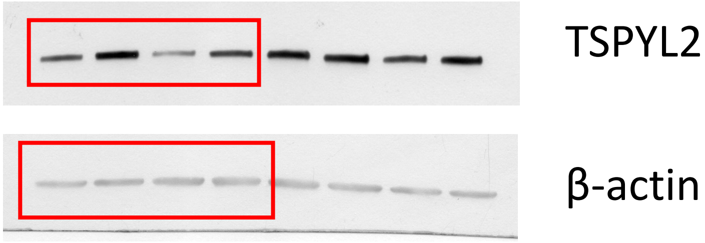

Fig. 3A

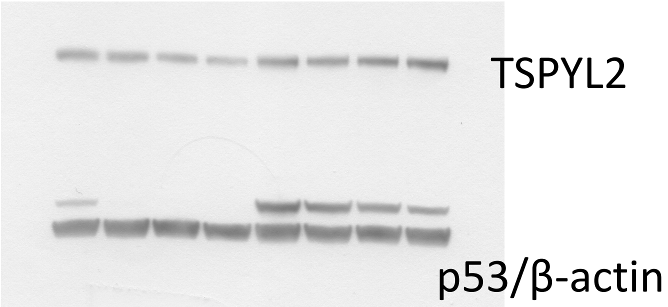

Fig. 3B

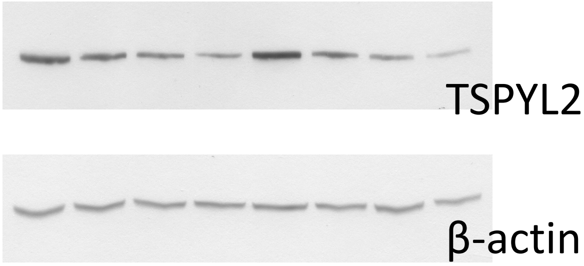

Fig. 3C

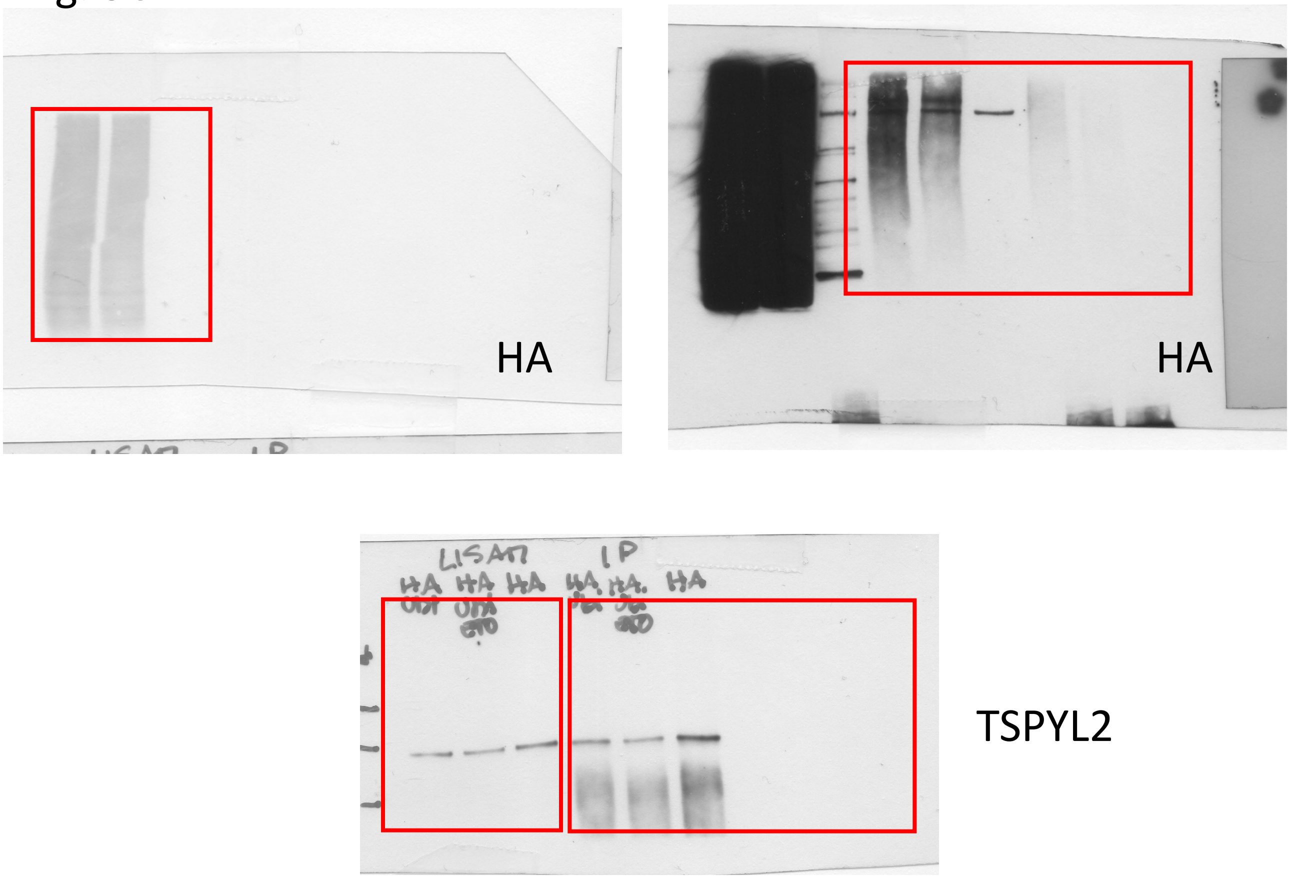

Fig. 3D

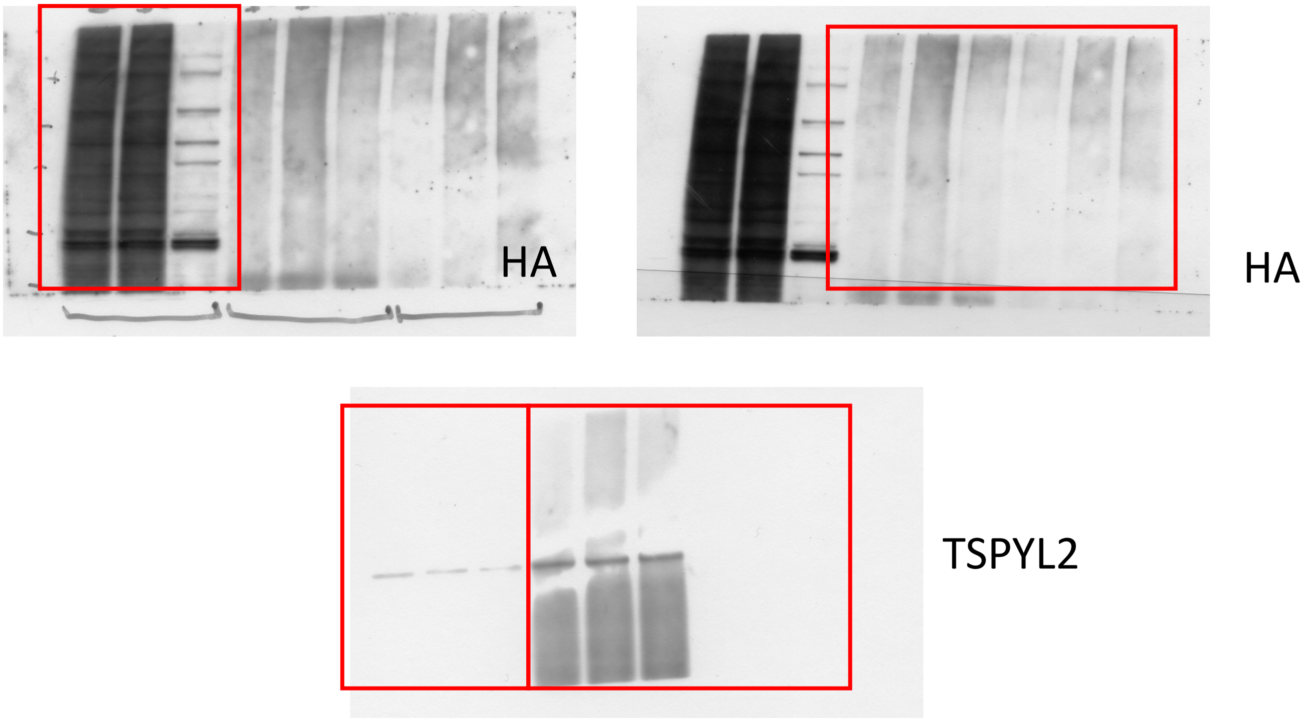

Fig. 3E

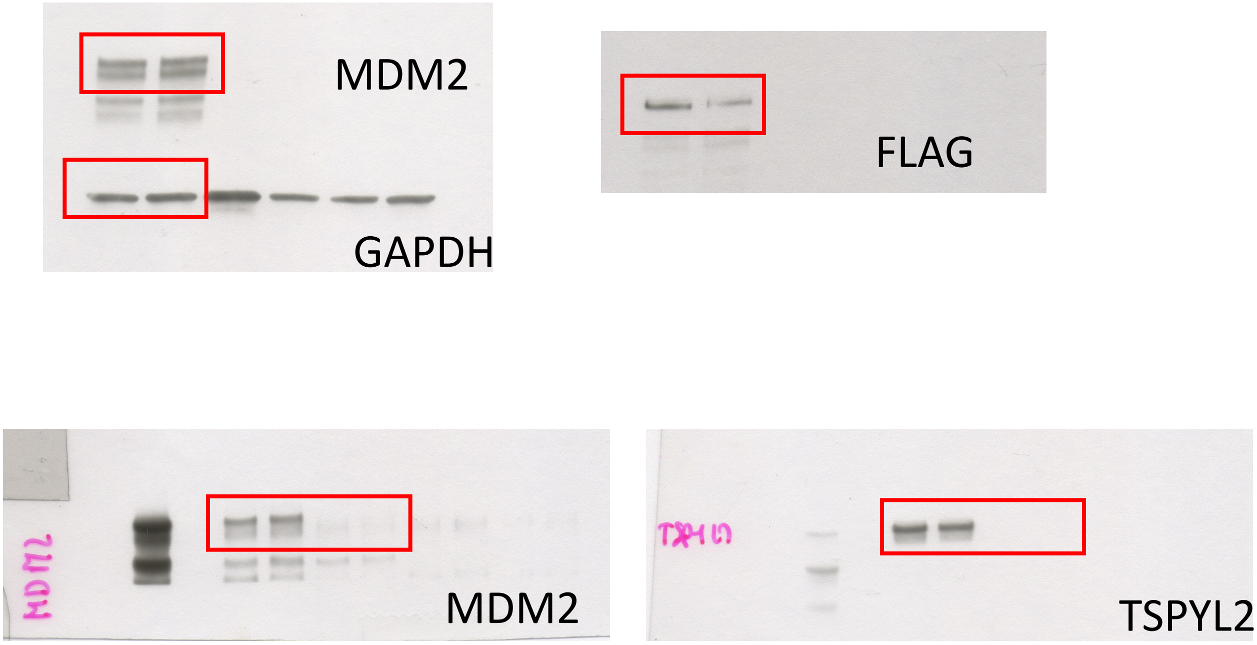

Fig. 3F

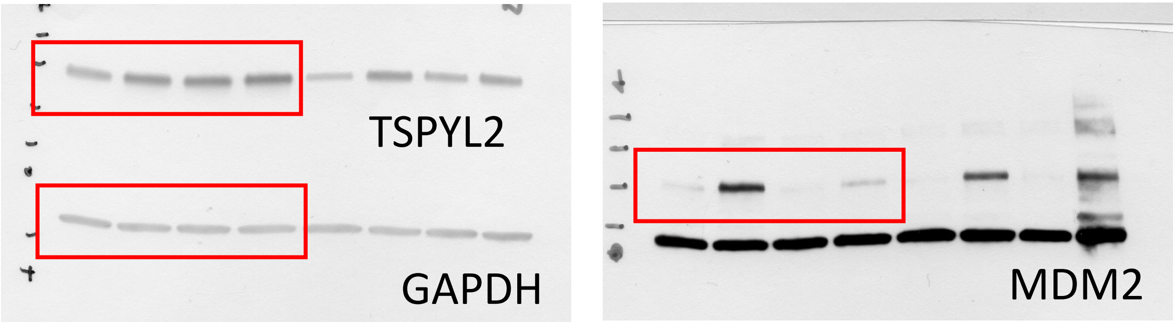

Fig. 3G

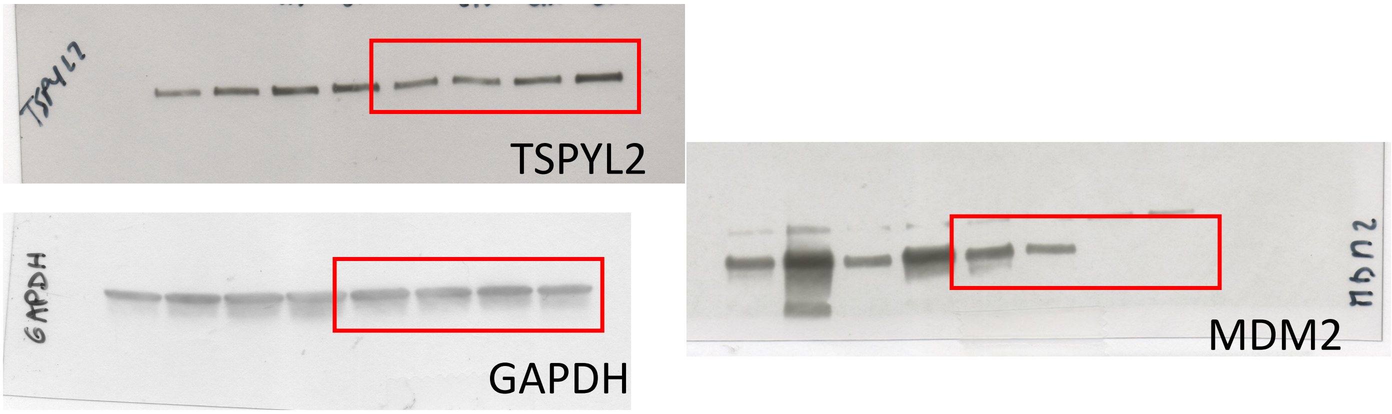

Fig. 3H

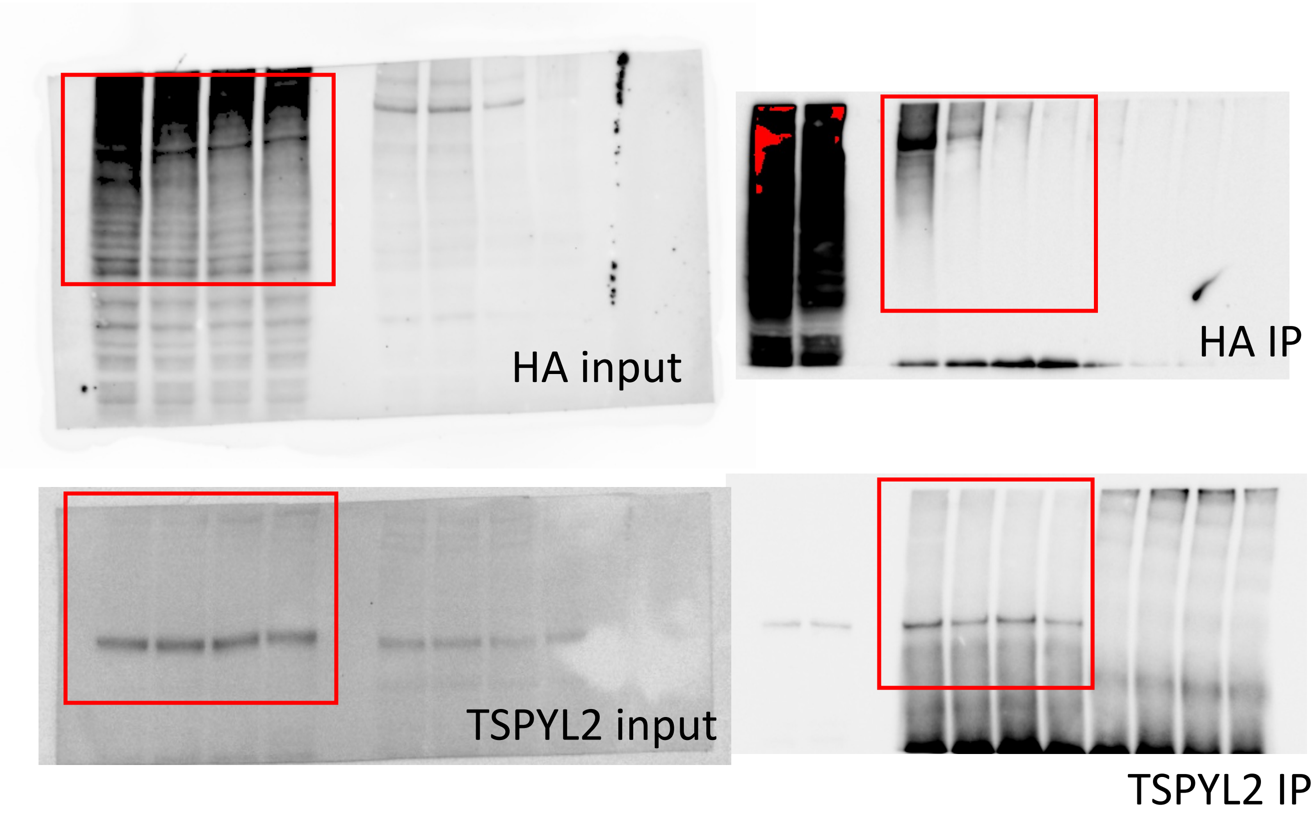

Fig. 3I

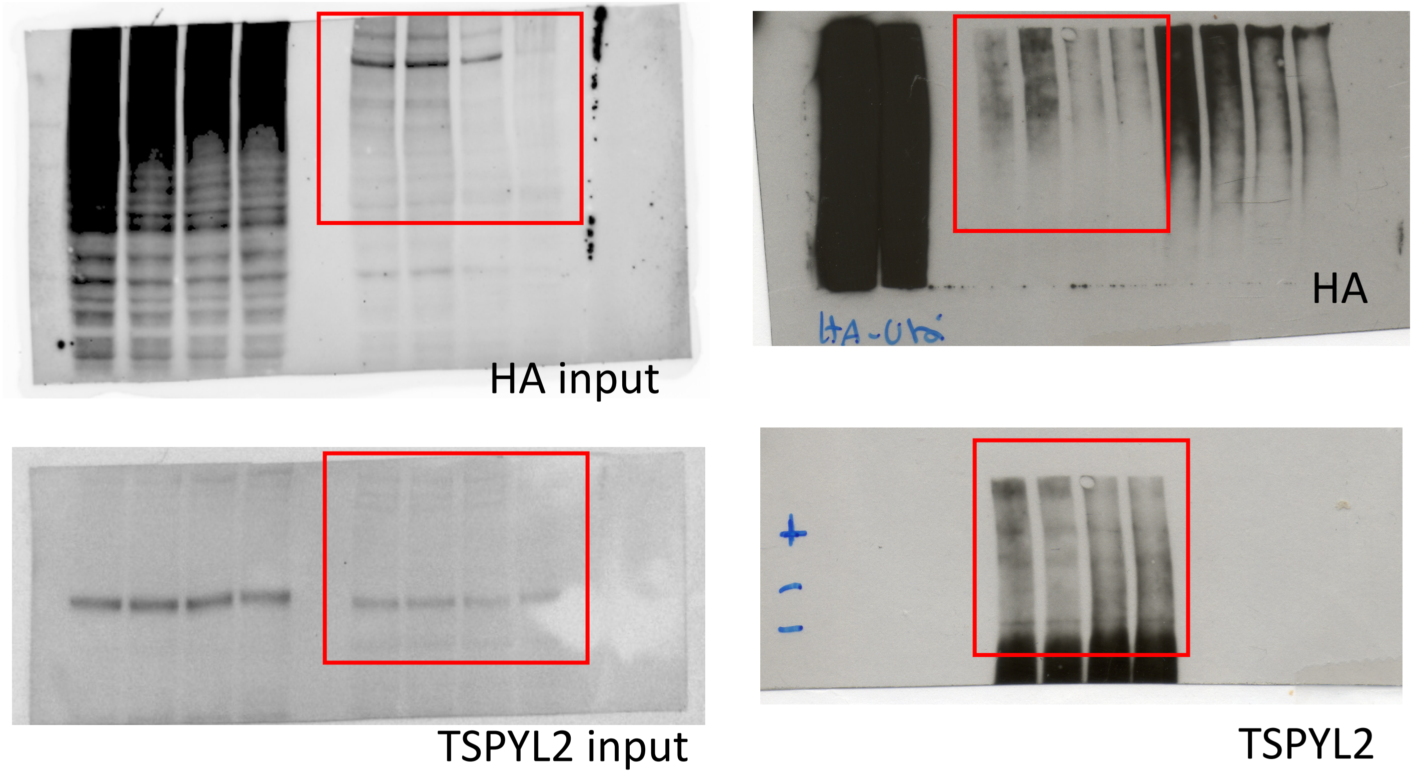

Fig. 4B

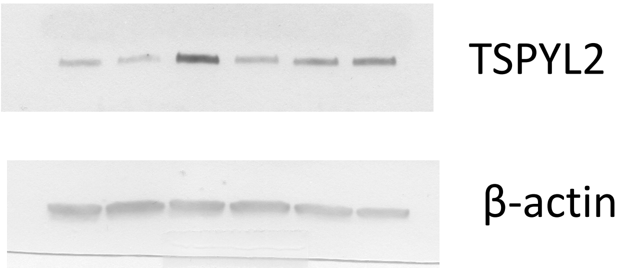

Fig. 4C

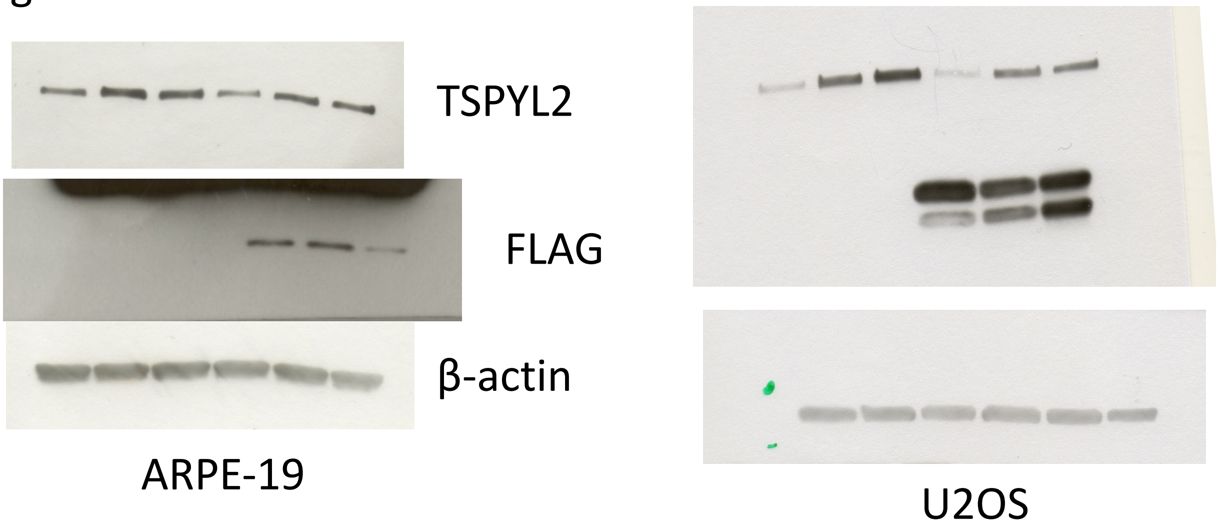

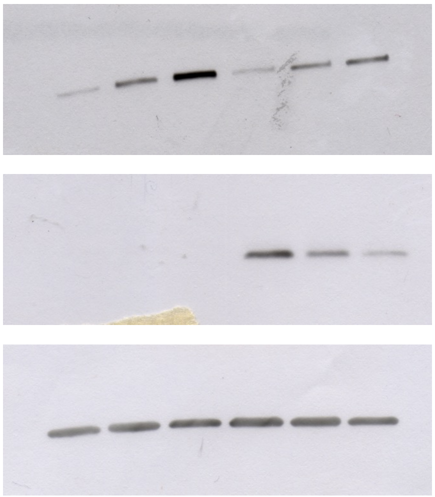

TSPYL2

FLAG

β-actin

HeLa

Fig. 4D

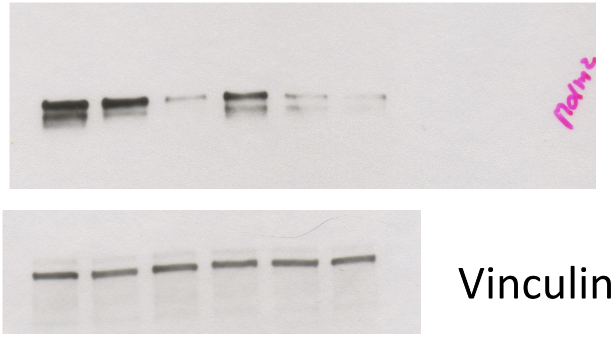

MDM2

Vinculin

Supplementary Fig. 1A

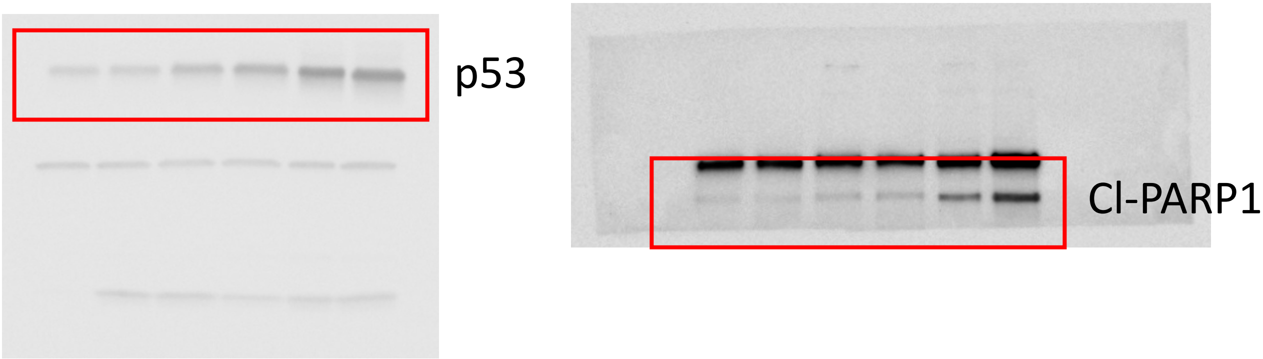

p53

Cl-PARP1

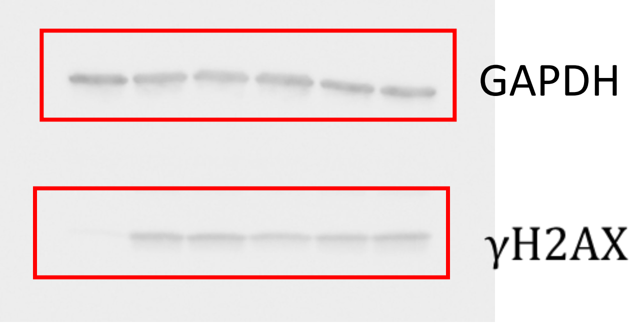

Supplementary Fig. 1E

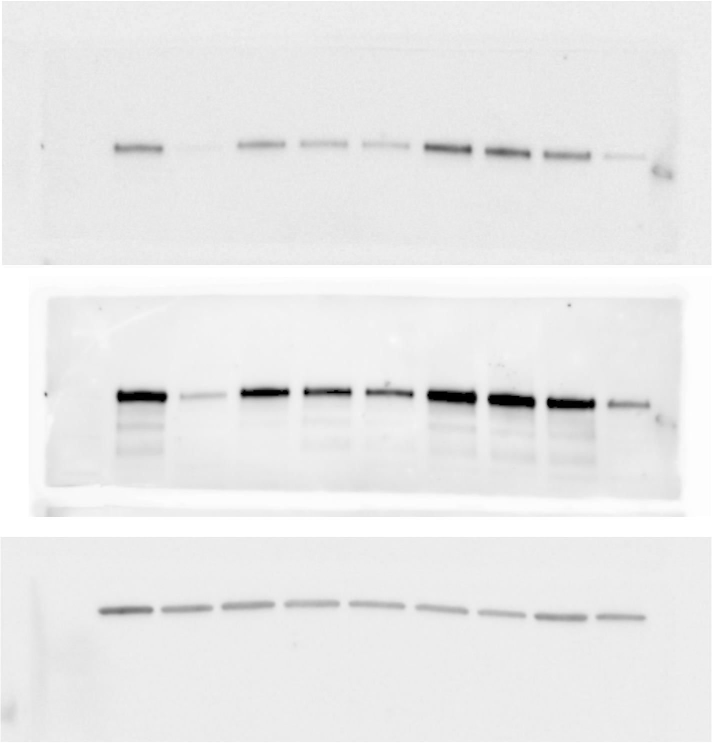

Supplementary Fig. 2C

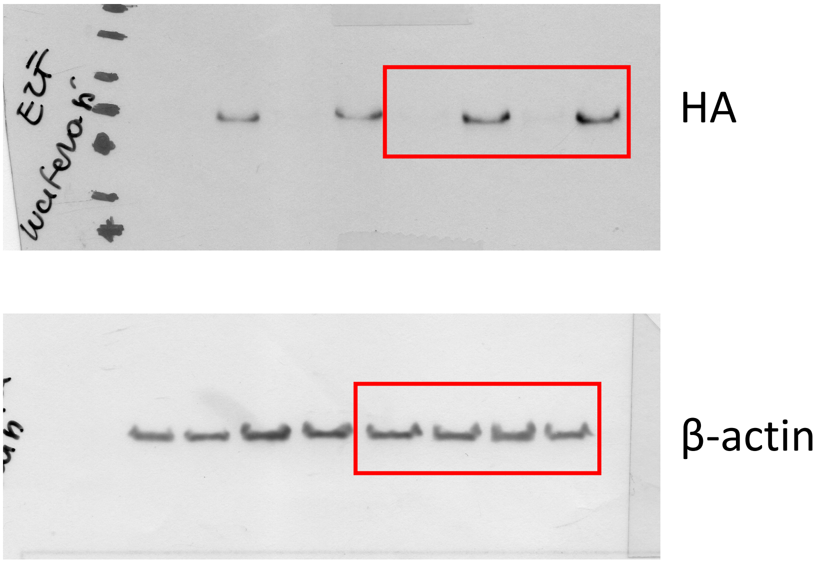

Supplementary Fig. 3

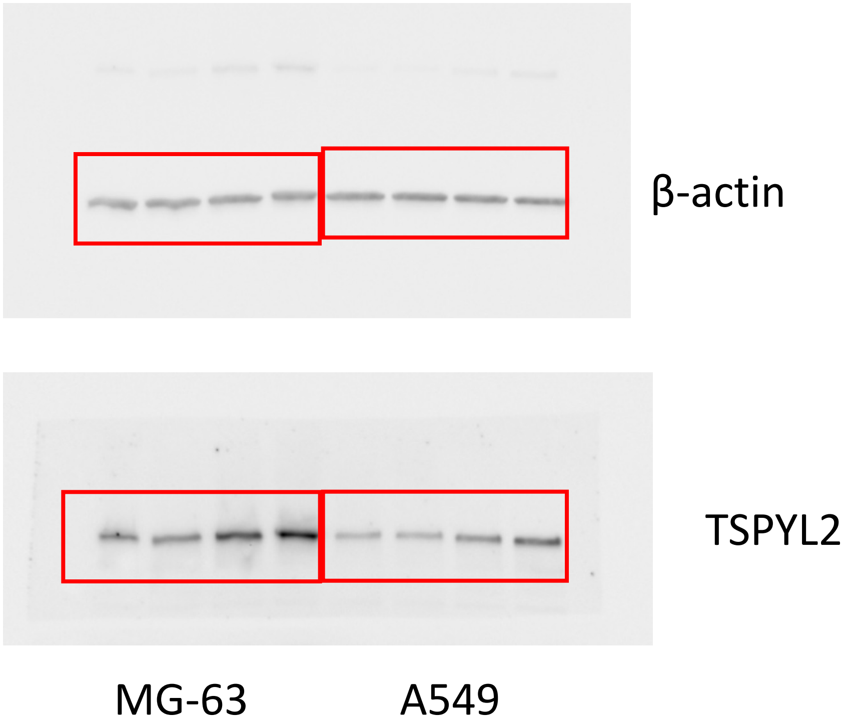

Supplementary Fig. 4

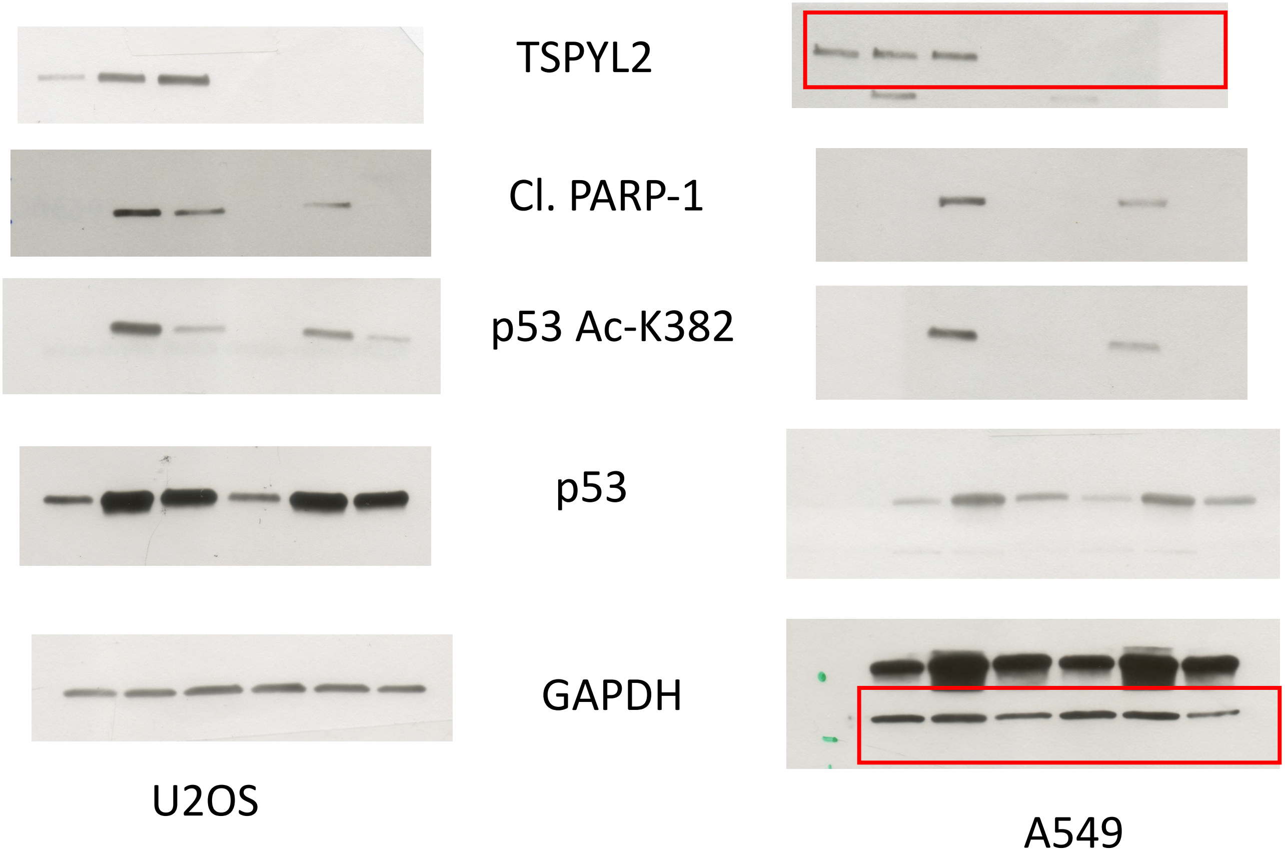

Supplement: Supplementary file 2 — Uncropped western blot [file 41419_2023_5722_MOESM2_ESM.pdf]
